# CCNB1IP1 prevents ubiquitination‐mediated destabilization of MYCN and potentiates tumourigenesis of *MYCN*‐amplificated neuroblastoma

**DOI:** 10.1002/ctm2.1328

**Published:** 2023-07-17

**Authors:** Yang Zhou, Hui Yan, Qiang Zhou, Penggao Wang, Fang Yang, Ziqiao Yuan, Qianming Du, Bo Zhai

**Affiliations:** ^1^ Henan Provincial Clinical Research Center for Pediatric Diseases, Henan Key Laboratory of Pediatric Genetics and Metabolic Diseases, Children's Hospital Affiliated to Zhengzhou University, Henan Children's Hospital Zhengzhou Children's Hospital Zhengzhou China; ^2^ Department of Cardiothoracic Surgery, Children's Hospital Affiliated to Zhengzhou University, Henan Children's Hospital Zhengzhou Children's Hospital Zhengzhou China; ^3^ Department of Pathology, Children's Hospital Affiliated to Zhengzhou University, Henan Children's Hospital Zhengzhou Children's Hospital Zhengzhou China; ^4^ School of Pharmaceutical Sciences Zhengzhou University Zhengzhou China; ^5^ General Clinical Research Center Nanjing First Hospital Nanjing Medical University Nanjing P. R. China; ^6^ School of Basic Medicine & Clinical Pharmacy China Pharmaceutical University Nanjing P. R. China

**Keywords:** cyclin B1 interacting protein 1, F box/WD‐40 domain protein 7, MYCN amplification, neuroblastoma, ubiquitination

## Abstract

**Background:**

*MYCN* amplification as a common genetic alteration that correlates with a poor prognosis for neuroblastoma (NB) patients. However, given the challenge of directly targeting MYCN, indirect strategies to modulate MYCN by interfering with its cofactors are attractive in NB treatment. Although cyclin B1 interacting protein 1 (CCNB1IP1) has been found to be upregulated in MYCN‐driven mouse NB tissues, its regulation with MYCN and collaboration in driving the biological behaviour of NB remains unknown.

**Methods:**

To evaluate the expression and clinical significance of CCNB1IP1 in NB patients, public datasets, clinical NB samples and cell lines were explored. MTT, EdU incorporation, colony and tumour sphere formation assays, and a mouse xenograft tumour model were utilized to examine the biological function of CCNB1IP1. The reciprocal manipulation of CCNB1IP1 and MYCN and the underlying mechanisms involved were investigated by gain‐ and loss‐of‐function approaches, dual‐luciferase assay, chromatin immunoprecipitation (CHIP) and co‐immunoprecipitation (Co‐IP) experiments.

**Results:**

CCNB1IP1 was upregulated in *MYCN*‐amplified (*MYCN*‐AM) NB cell lines and patients‐derived tumour tissues, which was associated with poor prognosis. Phenotypic studies revealed that CCNB1IP1 facilitated the proliferation and tumourigenicity of NB cells in cooperation with MYCN in vitro and in vivo. Mechanistically, MYCN directly mediates the transcription of CCNB1IP1, which in turn attenuated the ubiquitination and degradation of MYCN protein, thus enhancing CCNB1IP1‐MYCN cooperativity. Moreover, CCNB1IP1 competed with F box/WD‐40 domain protein 7 (FBXW7) for MYCN binding and enabled MYCN‐mediated tumourigenesis in a C‐terminal domain‐dependent manner.

**Conclusions:**

Our study revealed a previously uncharacterized mechanism of CCNB1IP1‐mediated MYCN protein stability and will provide new prospects for precise treatment of *MYCN*‐AM NB based on MYCN‐CCNB1IP1 interaction.

## Graphical abstract

1. CCNB1IP1 was overexpressed in *MYCN*‐amplified NB and was associated with poor prognosis.

2. CCNB1IP1 facilitated *MYCN*‐driven proliferation and tumourigenesis of NB cells.

3. MYCN directly activated CCNB1IP1 transcription, which in turn stabilized MYCN by competing with FBXW7 for MYCN binding in a C‐terminal domain‐dependent manner.

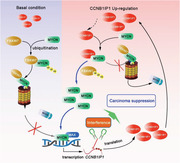

## 1 BACKGROUND

Neuroblastoma (NB) are tumours in the sympathetic nervous system that originate from pluripotent migratory neural crest cells where NB patients have higher mortality rates compared to other pediatric tumours.^1^, ^2^ The biological and clinical behaviour of NB is highly heterogeneous, characterized by spontaneous regression and curability to treatment‐resistant multi‐organ metastases, which are partly attributed to certain genetic alterations.^2^ Of these genetic alterations, amplification of the proto‐oncogene *MYCN* occurs in approximately 25% of NB patients, which often leads to a poor prognosis.^3^, ^4^ Although multimodal treatment including surgery, myeloablative chemotherapy, radiotherapy and immunotherapy have benefited low‐ and medium‐risk patients with long‐term overall survival (OS) rates above 80%, outcomes for high‐risk patients, especially those with *MYCN* amplification, remain unsatisfactory with 5‐year OS rates below 50%.^5^, ^6^ Therefore, investigating the mechanism that leads to MYCN‐driven malignant progression of NB and formulating a treatment strategy for high‐risk NB with *MYCN* amplification has become a crucial issue in the field of NB treatment.

MYCN was once considered a consensus target oncogene molecule for the treatment of high‐risk NB due to its multiple biological functions in NB, such as cell proliferation, metastasis, differentiation and death.^2^, ^3^, ^4^, ^7^ Yet, MYCN has neither a pocket structure that can be bound by small molecules nor enzyme activities that can be inhibited, rendering it almost impossible to be a direct target. Alternatively, indirect strategies that interfere with the transcription, translation, protein stability and target gene transcription of MYCN offer a bright future for targeting *MYCN*‐AM NB.^8^ Some target genes of MYCN have been found to assist MYCN‐driven survival and malignant transformation of NB cells, although they appear to be unnecessary for *MYCN* non‐amplified (*MYCN*‐NA) NB cells.^9^, ^10^ Furthermore, some MYCN target gene products have been found to directly or indirectly enhance the stability of MYCN proteins and thus synergize with MYCN to produce oncogenic effects, such as sirtuin 1 (SIRT1), Aurora kinase A (AURKA), proliferation‐associated 2G4 (PA2G4) and TEA domain‐4 (TEAD4).^10^, ^11^, ^12^, ^13^ These genes can also be targets for attacking *MYCN*‐AM NB. However, the available targets for the treatment of high‐risk NB with *MYCN* amplification are still limited. Nevertheless, there is still an urgent need to identify molecules with potential interactions and functional reciprocal support with MYCN as candidate targets for therapeutic applications. Therefore, identifying and targeting potential key survival factors modulated by MYCN will undoubtedly have far‐reaching implications for improving the treatment of high‐risk NB. Based on this reasoning, genes upregulated in *MYCN*‐AM NB were explored using multiple transcriptomic datasets of NB and in various cell lines. Therein, cyclin B1 interacting protein 1 (CCNB1IP1) positively correlated with MYCN expression in NB samples and cell lines. Moreover, CCNB1IP1 was related to poor long‐term survival in NB patients.

CCNB1IP1 was initially identified as an interacting protein of cyclin B1 involved in regulating cell cycle progression,^14^ and may be implicated in tumour events. CCNB1IP1 was found to be a component of HMG1C gene translocation fusion in uterine leiomyoma,^15^ and overexpressed in metastatic melanoma and hepatocellular carcinoma.^16^, ^17^ However, there are some contradictory reports as well. Studies have confirmed that CCNB1IP1 is under‐expressed in colon cancer, breast cancer and non‐small cell lung cancer.^16^ Additionally, it has a negative effect on cellular spreading, motility and invasion, but is required for cellular proliferation in the gastric cancer cell U2OS and breast cancer cell MCF‐7.^18^ Notably, we noticed a significant upregulation of CCNB1IP1 in tumour tissues derived from an MYCN‐driven NB mouse model of recurrent tumours in the study by Chesler L et al.^19^ However, the molecular basis regarding the effect of CCNB1IP1 on tumourigenicity, especially on *MYCN*‐AM NB, remains elusive and warrants further investigation.

In this present study, we found that CCNB1IP1 was overexpressed in *MYCN*‐AM NB samples and various cell lines, and was associated with the poor prognosis in NB patients. Silencing of CCNB1IP1 inhibited the proliferation and growth of *MYCN*‐AM NB cells in vitro and in vivo whereas overexpression of CCNB1IP1 had the opposite effect. Vitally, as a target gene of MYCN, CCNB1IP1 reciprocally stabilized the MYCN protein, forming an MYCN‐CCNB1IP1 positive feedback regulatory loop. Degradation of MYCN is known to be mediated mainly through the F‐box and WD‐40 domain protein 7 (FBXW7) E3 ligase pathway.^20^, ^21^ Mechanically, we found that CCNB1IP1 disrupted FBXW7‐dependent MYCN degradation by competing with FBXW7 for MYCN binding and promoted oncogenicity in a C‐terminal domain‐dependent manner. Our study provides rationality for CCNB1IP1 as a therapeutic candidate target for MYCN‐amplified high‐risk NB patients. Our finding also provides plausibility for predicting the risk of tumour progression and provides additional insight for drug development and refinement of individualized treatment regimens for high‐risk NB based on MYCN and CCNB1IP1 protein interactions.

## 2 METHODS

### 2.1 Cell lines and culture

Human NB cell lines SK‐N‐BE (2), SK‐N‐SH and SH‐SY5Y and HEK293T were obtained from the Cell Bank of the Chinese Academic of Sciences. SK‐N‐AS, IMR‐32 and BE(2)M17 were purchased from FuHeng biology (Shanghai, China). SK‐N‐SH and IMR‐32 were cultured in MEM medium with 10% FBS. SK‐N‐BE (2), SH‐SY5Y and BE(2)M17 were cultured in DMEM/F‐12 medium with 10% FBS. SK‐N‐AS and HEK293T were cultured in DMEM medium with 10% FBS. All cell lines were authenticated by short tandem repeat profiling analysis performed at Biowing Applied Biotechnology Co., Ltd. and maintained according to the manufacturer's instructions and cultured in a humidified incubator containing 5% CO_2_ at 37°C.

### 2.2 Reagents and antibodies

MTT (purity ≥98%) was purchased from Sigma‐Aldrich (88417). Cycloheximide (CHX, purity ≥99.81%) was purchased from MedChemExpress (HY‐12320). MG132 was purchased from Selleck Chemicals Inc (S2619). AceQ qPCR SYBR Green Master Mix was purchased from Vazyme. First‐strand cDNA Synthesis super Mix kit, RIPA buffer and TRIzol reagent were obtained from Sparkjade Biotech. Endotoxin‐free plasmid small extraction medium extraction kit was purchased from Tiangen. Immunohistochemistry (IHC) kits were purchased from Vector Laboratories. Dual Luciferase Reporter Gene Assay Kit, CHIP kit, EdU adulteration assay kit and Lipo8000 transfection reagent were purchased from Beyotime Biotech. Fetal bovine serum was purchased from Sigma‐Aldrich. Serum‐free lyophilization solution, rapid blocking solution, and universal antibody dilutions were purchased from New Cell & Molecular Biotech. IP/CoIP Kit (Magnetic Beads) was purchased from Dia‐An Biotech, Inc (Wuhan, China). The primary antibodies we used are shown in Table S1. Fluorescent secondary antibodies were purchased from Keygen Biotech (Shanghai, China).

### 2.3 RNA isolation, qRT‐PCR

Total RNA was extracted, reverse transcribed and subjected to PCR as previously described.^22^ Primer sequences for qRT‐PCR analysis are listed in Table S2. β‐actin expression was used for normalization. Relative expression of target genes was normalized using the internal control β‐actin and calculated using the 2^−ΔΔCq^ method.

### 2.4 Immunofluorescence (IF) assay

The cells were inoculated in a certain number into a special culture dish for inverted fluorescence microscopy, and the cells were washed twice with PBS and fixed in 4% paraformaldehyde when they were close to growing into a monolayer. The cells are permeabilized prior to incubation with the antibody. Cells are then blocked and incubated with the specific primary antibody overnight at 4°C. After washing with PBST the cells were incubated with a fluorescent secondary antibody at room temperature and protected from light. IF images of the cells were then taken under an inverted fluorescent microscope or laser confocal. Quantification of fluorescence intensity and co‐localization of fluorescent foci using ImageJ and the plugin JACoP. Mander's coefficients were employed to quantify the co‐localization between CCNB1IP1 and MYCN. This coefficient represents the proportion of the overlap between two fluorescent molecules, as previously described.^23^

### 2.5 Protein preparation and IB assay

NB cells or patient‐derived tissues placed on the ice were lysed with RIPA lysate containing 1 mM PMSF. Protein concentrations in the obtained lysates were assessed using the BCA method. The denatured proteins were separated by SDS‐PAGE and transferred to polyvinylidene fluoride membranes (Millipore, USA). After closure with Rapid Closure Solution, the membranes were incubated overnight at 4°C using the appropriate primary antibody. Following washing with PBST, the membranes were incubated for 1−2 hours at 37°C using the appropriate secondary antibody and washed again with PBST. Protein signals were visualized under a Bio‐Rad gel imaging system using ECL chromogenic reagents. β‐actin was used as a loading control.

### 2.6 Co‐IP assay

Co‐IP assay was performed using the IP/CoIP Kit (Magnetic Beads) according to the manufacturer's instructions. Briefly, cell lysates were prepared and collected. The lysates were incubated with magnetic beads coupled with antibodies to target proteins, generating the binding of the antigen to the antibody‐magnetic bead complex. The magnetic beads were separated and, the appropriate amount of loading buffer was added to which the sample was boiled at 95°C for 5 min, for sample elution. The eluted samples were subjected to SDS‐PAGE and IB assay to detect specific proteins.

### 2.7 Protein half‐life detection

The stability of the MYCN protein was examined in knockdown cells and cells with overexpression. CHX‐chase analysis was performed to determine the protein half‐life of MYCN. CHX treatment inhibited intracellular protein synthesis, thereby facilitating accurate detection of protein degradation. Briefly, in the MYCN half‐life assay, the target cells were incubated with CHX (10 μg/ml) for the indicated time, and then the cells were harvested and the relative protein expression of MYCN was measured by IB analysis.

### 2.8 Dual‐luciferase reporter assay

CCNB1IP1 promoter sequence wild‐type and mutant constructs were transfected with the internal control vector Renilla into specifically treated NB cells according to the manufacturer's instructions. The sequence of CCNB1IP1 promoter sequence is provided in Table S3. The luciferase assays were performed using the Dual‐Luciferase Reporter Gene Assay Kit (Beyotime Biotech, Hangzhou, China) as previously described.^24^ The luciferase activity was normalized to Renilla expression for each sample.

### 2.9 CHIP assay

CHIP was performed according to the manufacturer's instructions. Briefly, cells transfected with specific silencing or overexpression plasmids were collected, cross‐linked and sonicated. IP assay was performed with an anti‐MYCN antibody co‐incubated with the lysis products. IgG was used as a loading control. Immunoprecipitation complexes were obtained and purified, and the binding of MYCN to the CCNB1IP1 promoter was detected by semi‐quantitative PCR. The primers used to amplify the putative binding CCNB1IP1 promoter fragment are listed in Table S4.

### 2.10 Colony formation assay

The procedure was performed as previously described.^24^ NB cells stably expressing or silencing specific genes were seeded into six‐well plates at 500 cells per well and cultured for approximately 2 weeks. At the end of the culture, the medium was discarded and the cells were washed three times with cold sterile 1×PBS, fixed in 4% formaldehyde and stained with 0.1% crystal violet. The colonies' number was then counted macroscopically.

### 2.11 EdU incorporation assay

EdU incorporation assay was performed according to the manufacturer's instructions. Briefly, the target cells transfected with CCNB1IP1 overexpression plasmids or shRNA were seeded into 96 well plates at 5000 cells per well. Cells were fixed with 4% neutral paraformaldehyde and permeabilized with 0.5% Triton X‐100 following incubation with EdU. Click EdU reactions were performed and the cells were placed under an inverted fluorescent microscope for observation and photography.

### 2.12 Tumor sphere formation and live/dead cells detection

Cells grown to the logarithmic phase were taken, washed with PBS, trypsin digested, centrifuged and resuspended. Cells were seeded at the required amount into ultra‐low adherence 96‐well culture plates for several days. To determine the viability of spheroid‐forming cells, a combination of calcein acetoxymethyl ester (Calcein AM) and propidium iodide (PI) was used. Following staining the cell spheroids were subjected to inverted fluorescence microscopy for fluorescence imaging where Calcein AM stained live cells (green) and PI stained dead cells (red).

### 2.13 Ubiquitination analysis

Target cells were transfected with plasmids that expressed or silenced specific proteins, and the protein lysates were then collected and prepared. IP assay was performed with anti‐MYCN or anti‐HA‐MYCN antibodies. MYCN protein polyubiquitination inco‐IP products were subsequently determined by IB assay with anti‐ubiquitin primary antibody. The IgG group was set as a loading control.

### 2.14 Cell transfection

The shRNA targeting CCNB1IP1 (#1 TRCN0000003418 and #2 TRCN0000003419) and MYCN (#1 TRCN0000020695 and #2 TRCN0000020698) in pLKO.1 lentiviral vector was purchased from Millipore Sigma (St. Louis, MO) and used as recommended by the manufacturer. The packaging plasmids, PsPAX2 and pMD2.G were purchased from the MiaoLing Plasmid Sharing Platform. Targeting sequences of shRNA for FBXW7, E3 ubiquitin ligase tripartite motif protein 32 (Trim32), Huwe1, Ubiquitin carboxyl‐terminal hydrolase 3 (USP3) or Ubiquitin carboxyl‐terminal hydrolase 5 (USP5) are listed in Table S5. The Flag‐tagged coding sequence of CCNB1IP1 WT or the relevant M1‐M4 mutants, HA‐tagged MYCN WT or the relevant MYCN^48‐89^ and MYCN^△48‐89^ mutants, Myc‐tagged FBXW7 WT or the FBXW7^△F‐box^ mutant were cloned into the lentiviral vector, pCDH‐CMV‐MCS‐EF1‐GFP+Puro (Changsha Youze Biotechnology Co., Ltd) to generate expression plasmids. The relevant lentiviral particles were generated in HEK293T cells using the Lentiviral Packaging Kit according to the manufacturer's instructions. As for cell transfection, the target cells were transfected with lentiviral‐encoded target DNA constructs using lipo8000 transfection reagent according to the manufacturer's instruction and stably transfected cells were selected with puromycin (1 μg/ml).

### 2.15 Protein‐protein docking

Preparation of protein structure: CCNB1IP1, MYCN target protein structures were predicted by I‐TASSER online server (https://zhanggroup.org//I‐TASSER/). All protein structures were treated in a molecular manipulation environment (MOE 2019.1) including removal of water and ions, protonation, addition of missing atoms and complement of missing groups, and protein energy minimization. Molecular docking: the processed CCNB1IP1 and MYCN proteins were introduced into the receptor and ligand modules of HDOCK software, respectively, and the docking sites were selected to be the entire surface of the protein. The conformation of the protein‐protein complex was set to 100. A hybrid algorithm for template‐based and template‐free Docking automatically predicted its interaction, which was evaluated using a Docking Score and RMSD of the ligand. Docking results were visualized with Pymol2.1 software.

### 2.16 Animal procedures

Balb/c male nude mice purchased from Vitone River Laboratory Animal Technology Co., Ltd. were housed in a standard SPF animal room equipped with sealed air filtration devices. All experimental procedures on mice were approved by the Animal Ethics Committee of Zhengzhou University and performed in accordance with the institutional guidelines (No. ZZUIRB2021‐99). The mice were randomly grouped and subjected to a period of acclimatization prior to the experiment. Mice were injected with IMR‐32 cells (200 μl, 2 × 10^6^ cells) stably transfected with non‐targeted shRNA (shCtrl), CCNB1IP1‐targeted shRNA (shCCNB1IP1#1 and #2); empty vector (Vector) or CCNB1IP1 overexpression plasmid; MYCN‐targeted shRNA (shMYCN); or MYCN‐targeted shRNA combined with CCNB1IP1 plasmid (shMYCN+CCNB1IP1) in the subcutaneous space to construct NB cells‐derived xenograft tumour models. For the tumour growth assay, subcutaneous tumour volumes were measured every 5 days, and mice were euthanized at the end of the experiment. The tumours were then peeled and weighed. The volume was calculated by the formula: V = (a × b^2^)/2, where **a** and **b** are the long axis and short axis of the tumour, respectively.

### 2.17 IHC detection

IMR‐32 cells‐derived xenogeneic tissues from nude mice or primary tumour tissues from untreated NB patients were obtained and fixed in 4% paraformaldehyde, dehydrated in an alcohol gradient, hyalinized in xylene, and paraffin‐embedded. IHC staining for CCNB1IP1, MYCN and ki67 expression was performed using the appropriate primary antibodies following the manufacturer's instructions for the IHC kit. All sections were photographed under a vertical microscope (Nikon, Japan). The sections were analyzed with a double‐blind method employing a semi‐quantitative scoring system as previously reported.^25^ The number of positive cells was divided into four grades 4 (> 75%), 3 (51%–75%), 2 (25%–50%), 1 (5%–25%) or 0 (<5%), while the intensity of staining was divided into three grades, 3 (dark brown), 2 (light brown), 1 (light yellow) or 0 (no colour). The total IHC score was generated by multiplying the two grade scores, defined as negative (≤3), weak (> 3 and ≤6), moderate (> 6 and ≤9), and strong (>9).

### 2.18 Histological sample

This study was approved by the ethics review committee of Zhengzhou University according to the standards and guidelines of the institutional review committee (No. 2021‐H‐K24). All samples involved in this study were untreated primary tumours from NB patients with defined *MYCN* amplification status. The patients signed written informed consent at the Children's Hospital Affiliated with Zhengzhou University upon admission. All studies on human samples were conducted in accordance with accepted ethical norms (Helsinki declaration, CIOMS, Belmont Report, American common rules).

### 2.19 Dataset analysis

RNA sequencing data for NB patients and cell lines and clinical follow‐up information for patients were downloaded from the Gene Expression Omnibus (GEO) database (https://www.ncbi.nlm.nih.gov/geo/, GEO accession numbers: GSE49710, GSE120572, GSE45547 and GSE80149) and the Therapeutically Applicable Research to Generate Effective Treatments (TARGET) data portal (https://ocg.cancer.gov/programs/target). NB patients and cell lines containing complete gene expression data and a defined MYCN amplification status (AM or NA) were included in this study following the filtering out of some samples with incomplete information. For the analysis of OS and event‐free survival (EFS), patients were divided into a CCNB1IP1 high expression group (top 25%) and a CCNB1IP1 low expression group (bottom 75%), and Kaplan‐Meier curves were created using an online genomic analysis visualization platform (https://r2platform.com).

### 2.20 Statistical analysis

Data obtained from at least three independent experiments were expressed as mean ± SD. Statistical analysis was performed using SPSS version 25.0. Student's t‐test and one‐way ANOVA were calculated to compare the differences between two groups or multiple groups, respectively. The *χ* ^2^ test was used to analyze the association between CCNB1IP1 expression and clinicopathologic variables. A statistically significant difference was shown as ^*^ *p* < 0.05, ^**^ *p* < 0.01 and ^***^ *p* < 0.001.

## 3 RESULTS

### 3.1 Expression of *CCNB1IP1* is correlated with the *MYCN* amplification and expression in NB samples and cell lines

**FIGURE 1:**
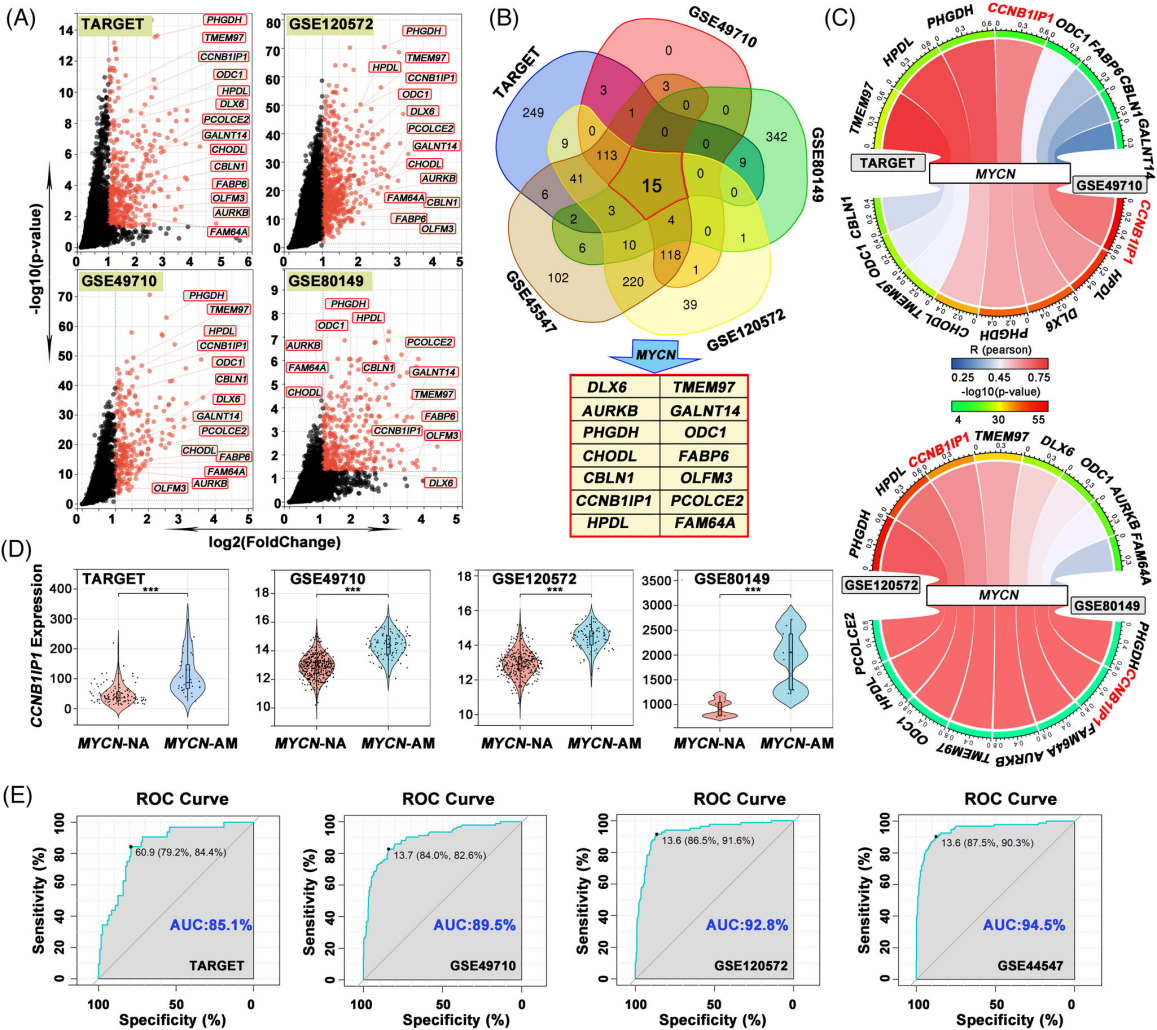
Expression of CCNB1IP1 in NB tissues and cell lines and its relationship with MYCN amplification and expression. (A) Differential genes between *MYCN*‐AM and NA NB samples or cell lines from TARGET, GEO (GSE49710, GSE120572 and GSE80149) datasets (log2FC > 1, FDR < 0.05). (B) Venn diagram of upregulated genes in *MYCN*‐AM NB samples or cell lines from these datasets above (vs. *MYCN*‐NA). Co‐upregulated genes in *MYCN*‐AM NB are marked with red boxes. (C) Correlation between *CCNB1IP1* and *MYCN* transcription levels in NB tissues and cells from public datasets. The values outside the outer circle and the colour of the inner circle represent the correlation coefficient R‐value, and the colour of the outer circle represents the log10 (*P*‐values). The correlation was tested by Pearson correlation analysis. (D) *CCNB1IP1* expression in *MYCN*‐NA and *MYCN*‐AM NB patients in the indicated datasets. (E) Receiver operating characteristic (ROC) curves showed the specificity of *CCNB1IP1* expression for reflection of *MYCN*‐AM subtypes in NB patients. AUC, the area under the curve.

In view of the “non‐druggability” of MYCN, attempts to tackle MYCN have been focused on indirect targeting strategies. In addition to MYCN itself, some specific downstream genes of MYCN are specifically required for *MYCN*‐AM NB growth.^9^, ^10^ To identify potential MYCN‐dependent pro‐survival genes, the publicly available transcriptome data from TARGET and GEO (GSE49710, GSE80149, GSE120572 and GSE45547) were obtained for analysis and differentially expressed genes between *MYCN*‐AM and *MYCN*‐NA NB samples or cell lines were compared (log_2_FC > 1, FDR < 0.05). As shown in Figure 1A,B and Figure S1A, a total of 15 shared differential genes, including *MYCN*, were identified from NB samples and cell lines. Correlation analysis revealed that *CCNB1IP1* was positively correlated with *MYCN* expression in *MYCN*‐AM NB samples and in the cell lines in all datasets mentioned above (Figure 1C and Figure S1B). Furthermore, a comparative analysis of these samples with or without *MYCN* amplification was performed. In the TARGET and GEO databases, CCNB1IP1 was identified to be highly expressed in *MYCN*‐amplified NB tissues and cell lines (Figure 1D and Figure S1C). To assess the specificity of *CCNB1IP1* expression for *MYCN* amplification in NB patients, receiver operating characteristic curves were performed. As shown in Figure 1E, the area under the curve (AUC) value in the TARGET database was 85.1%, and the AUC values in the GEO datasets (GSE49710, GSE120572 and GSE45547) were 89.5%, 92.8% and 94.5%, respectively. These results suggest that *CCNB1IP1* expression is enriched in *MYCN*‐AM NB and may represent a high‐risk factor for disease progression.

### 3.2 CCNB1IP1 is overexpressed in *MYCN*‐AM NB and is predictive of malignancy

**FIGURE 2:**
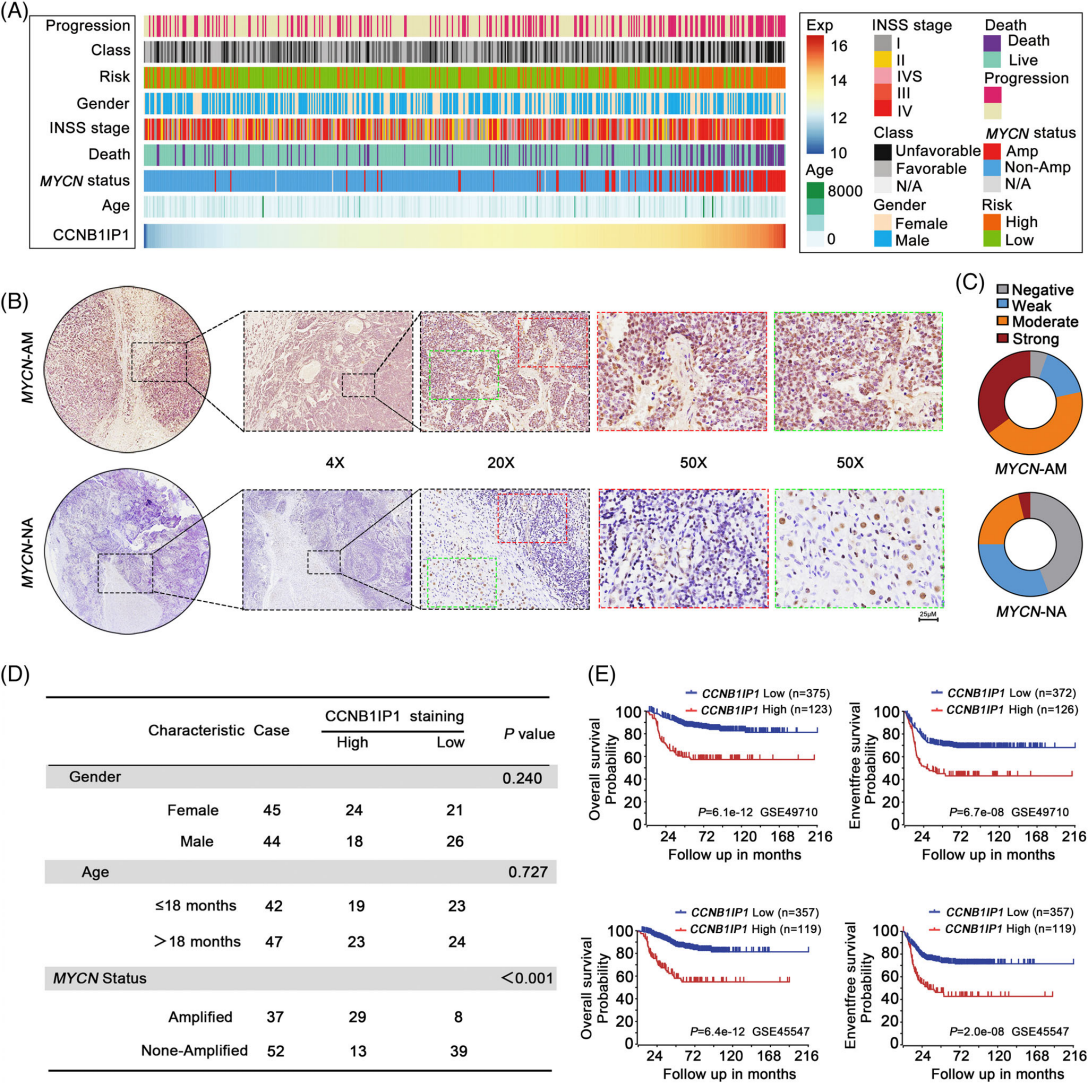
CCNB1IP1 expression was associated with poor clinical features in NB patients. (A) The landscape of CCNB1IP1‐related clinicopathological features of NB in the GSE49710 cohort. (B) IHC assay to detect CCNB1IP1 protein expression in *MYCN*‐AM and NA NB sample tissues. The dashed box represents the area to be amplified. Scale bar = 25 μm. (C) IHC score of CCNB1IP1 in *MYCN*‐AM and NA NB sample tissues. (D) Correlation of CCNB1IP1 expression with clinical characteristics of NB patients (age at diagnosis, gender and *MYCN* amplification status). (E) Kaplan‐Meier estimation of the OS and EFS based on the CCNB1IP1 transcriptome data from the GSE49710 and GSE45547 cohorts.

To explore the relationship between CCNB1IP1 expression and the clinical patterns and pathological features, we performed an analysis based on public databases. As shown in Figure 2A, CCNB1IP1 expression was positively associated with the unfavourable class (poor treatment responsiveness), high‐risk grade, disease‐related death and *MYCN* amplification status in NB patients. This demonstrates that patients with higher CCNB1IP1 expression exhibit poorer clinical patterns and pathological features. Furthermore, we examined the expression of CCNB1IP1 in samples obtained from NB patients with or without *MYCN* amplification. As shown in Figure 2B, CCNB1IP1 was overexpressed in NB patients with *MYCN* amplification compared to those with *MYCN* non‐amplification. High expression of CCNB1IP1(IHC score defined as moderate and strong) was observed in approximately 78.4% of *MYCN*‐AM NB patients, whereas low expression of CCNB1IP1 (IHC score defined as low and negative) was detected in about 75.0% of *MYCN*‐NA NB patients (Figure 2C). Furthermore, CCNB1IP1 expression was positively correlated with the *MYCN* amplification status (*p* < 0.001, *χ* ^2^ test), but not with gender (*p* = 0.240, *χ* ^2^ test) and age (*p* = 0.727, *χ* ^2^ test) (Figure 2D). To elucidate the relationship between CCNB1IP1 and *MYCN* amplification, protein levels of CCNB1IP1 and MYCN were detected by IB assay. As shown in Figure S2, the protein expression levels of CCNB1IP1 in NB cell lines were found to be significantly higher in *MYCN*‐AM cell lines (SK‐N‐BE(2), BE(2)M17 and IMR‐32) compared to NA cell lines (SK‐N‐AS, SH‐SY5Y and SK‐N‐SH). These findings from NB samples and cell lines are substantially consistent with the bioinformatics analysis from public datasets. Moreover, *CCNB1IP1* expression was identified to be closely associated with the prognosis of NB patients (https://r2platform.com), with the higher expression of *CCNB1IP1* associated with lower OS and EFS in NB patients (Figure 2E). The above underscores the connection between CNB1IP11 overexpression and *MYCN* amplification and the adverse prognosis of NB patients.

### 3.3 MYCN directly mediates the transcriptional activation of CCNB1IP1

**FIGURE 3:**
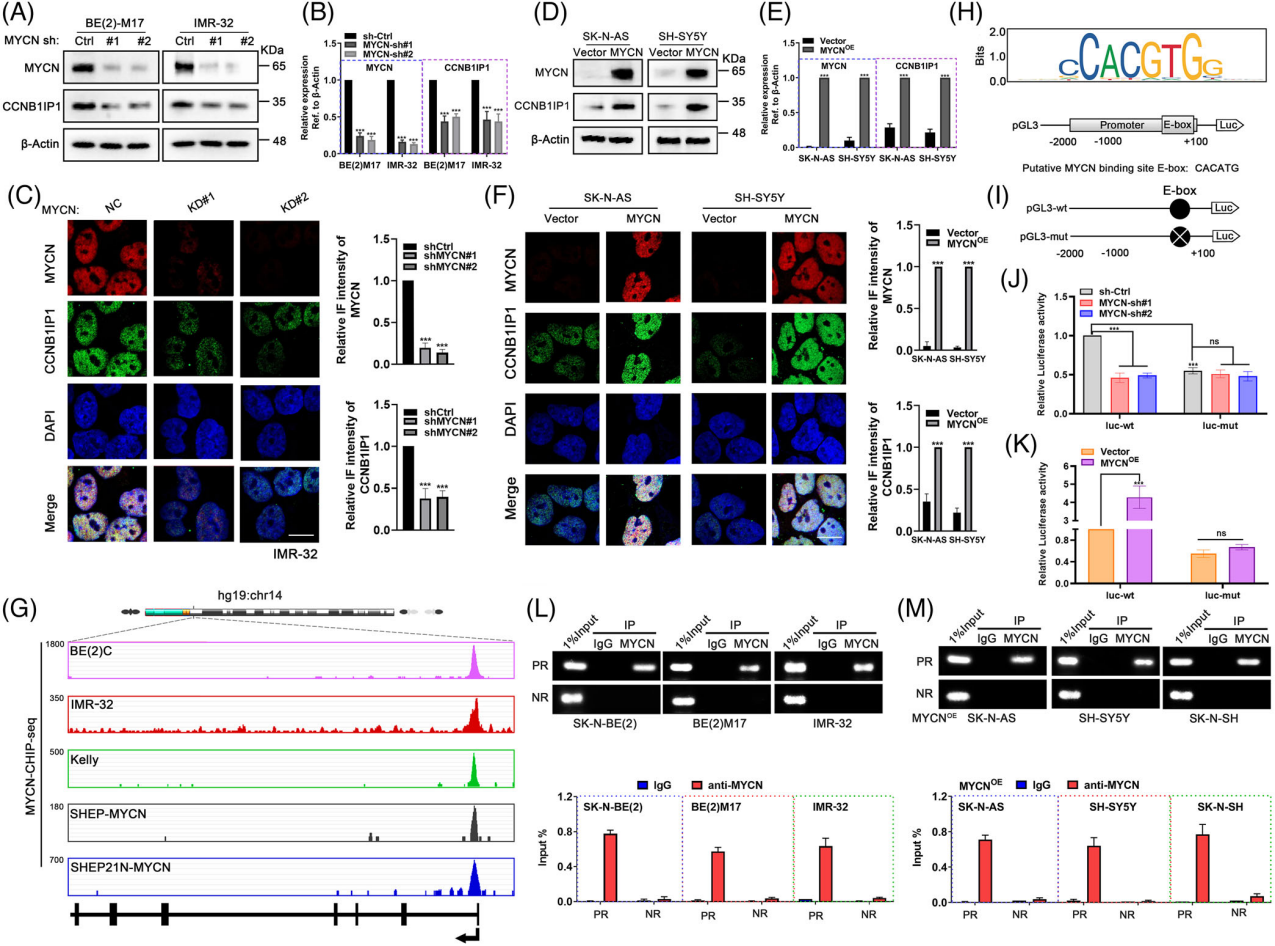
MYCN directly regulates the expression and transactivation of CCNB1IP1 in NB cells. (A) Immunoblot (IB) assay of MYCN and CCNB1IP1 expression in BE(2)M17 and IMR‐32 cells was performed upon MYCN‐shRNA knockdown for 48 h. (B) Quantification of A. (C) Representative IF images of IMR‐32 cells. MYCN, red; CCNB1IP1, green; DAPI, blue. Scale bar = 10 μm. (D) IB assay of MYCN and CCNB1IP1 expression was performed in SK‐N‐AS and SH‐SY5Y cells upon MYCN overexpression. (E) Quantification of D. (F) Representative IF images. MYCN, red; CCNB1IP1, green; DAPI, blue. Scale bar = 10 μm (G) ChIP‐Seq analysis shows the enrichment tracks of MYCN binding to the near promoter region of the CCNB1IP1 locus across a panel of NB cell lines. NB cell lines with MYCN amplified (BE(2)C, IMR‐32 and Kelly) and inducible MYCN systems (SHEP‐MYCN and SHEP21N‐MYCN). Data analysis from R2: Genomics Analysis and Visualization Platform (http://r2.amc.nl). (H) Predicted MYCN binding site on the CCNB1IP1 promoter (from −2000 to +100 bp upstream) using the Jaspar database (https://jaspar.genereg.net/). (I) Schematic representation of the putative MYCN binding site E‐box (AATCACATGGCC) and the mutation site (AATGTGATGGCC) on the CCNB1IP1 promoter. (J, K) Luciferase activity was assayed in MYCN‐silenced IMR‐32 cells or ‐overexpressed SK‐N‐AS cells transfected with WT or mutant pGL3‐CCNB1IP1 promoter constructs, and pRL‐TK was used as an internal control. ChIP assay manifested MYCN enrichment in the promoter region of CCNB1IP1 in *MYCN*‐AM NB cells (L) and *MYCN*‐NA NB cells with MYCN overexpression (M). PR: Putative binding region; NR: Negative binding region. Data represent the mean ± SD of at least three independent experiments (ns, no significant differences; ^**^ *p <* 0.01 and ^***^ *p <* 0.001).

Given the correlation between CCNB1IP1 expression and *MYCN* amplification or expression, we speculate that there may exist mutual regulatory relationships between them. MYCN as a classical oncogenic transcription factor is involved in the positive regulation of several oncogenes, such as *MDM2*, *MRP*, *E2F5* and *ALDH18A1*,^26^, ^27^, ^28^, ^29^, ^30^ thus we considered whether the expression of CCNB1IP1 was regulated by MYCN. To verify this, the two constructed shRNAs targeting MYCN with different sequences were transfected into *MYCN*‐AM cell lines and their knockdown efficiency was examined. As expected, the ablation of MYCN significantly reduced both the CCNB1IP1 mRNA and protein levels in *MYCN*‐AM NB cells (Figure 3A,B and Figure S3A,B). We noticed that the intracellular IF intensity of CCNB1IP1 was obviously diminished upon MYCN knockdown as well (Figure 3C and Figure S3C). To fully confirm the regulation of MYCN on CCNB1IP1 expression, we exogenously overexpressed MYCN in *MYCN*‐NA cell lines and found a significant increase in mRNA and protein expression as well as IF staining intensity of CCNB1IP1 (Figure 3D–F and Figure S3D–F). To explore whether MYCN directly modulated the transcriptional activation of CCNB1IP1, we explored the binding of MYCN on the CCNB1IP1 gene based on CHIP‐seq data in the R2: Genomics Analysis and Visualization Platform (http://r2.amc.nl). As shown in Figure 3G, high enrichment of MYCN in the nearby region of the CCNB1IP1 promoter was observed in a variety of *MYCN*‐amplified and constitutively overexpressing NB cell lines, as demonstrated by the peak signal. This suggests a potential regulatory role of MYCN in the expression of CCNB1IP1. Next, we predicted the potential motifs of the CCNB1IP1 promoter region for MYCN binding (https://jaspar.genereg.net/). As shown in Figure 3H, MYCN potentially interacts with the E‐box of the CCNB1IP1 promoter region, to which the reporter gene constructs containing the CCNB1IP1 promoter with wild‐type (WT) and the E‐box region mutant were established in order to confirm the transactivation of CCNB1IP1 by MYCN (Figure 3I). As shown in Figure 3J,K, the luciferase activity of the pGL3‐CCNB1IP1 promoter was significantly diminished or elevated in *MYCN*‐AM cells and *MYCN*‐NA cells upon silencing or overexpression of MYCN, respectively. Interestingly, the luciferase activity was significantly reduced upon E‐box mutation and was unaffected even by the gain or loss of MYCN expression. Moreover, enrichment of MYCN to the E‐box containing region of CCNB1IP1 promoter was detected in the co‐precipitated product of MYCN primary antibody by CHIP assay in *MYCN*‐AM NB cells or *MYCN*‐NA cells with MYCN exogenously introduced with MYCN (Figure 3L,M). The above results suggest that CCNB1IP1 is directly regulated at the transcriptional level as a downstream gene of MYCN.

### 3.4 CCNB1IP1 expression is essential for the proliferation and tumourigenicity of *MYCN*‐AM NB cells

**FIGURE 4:**
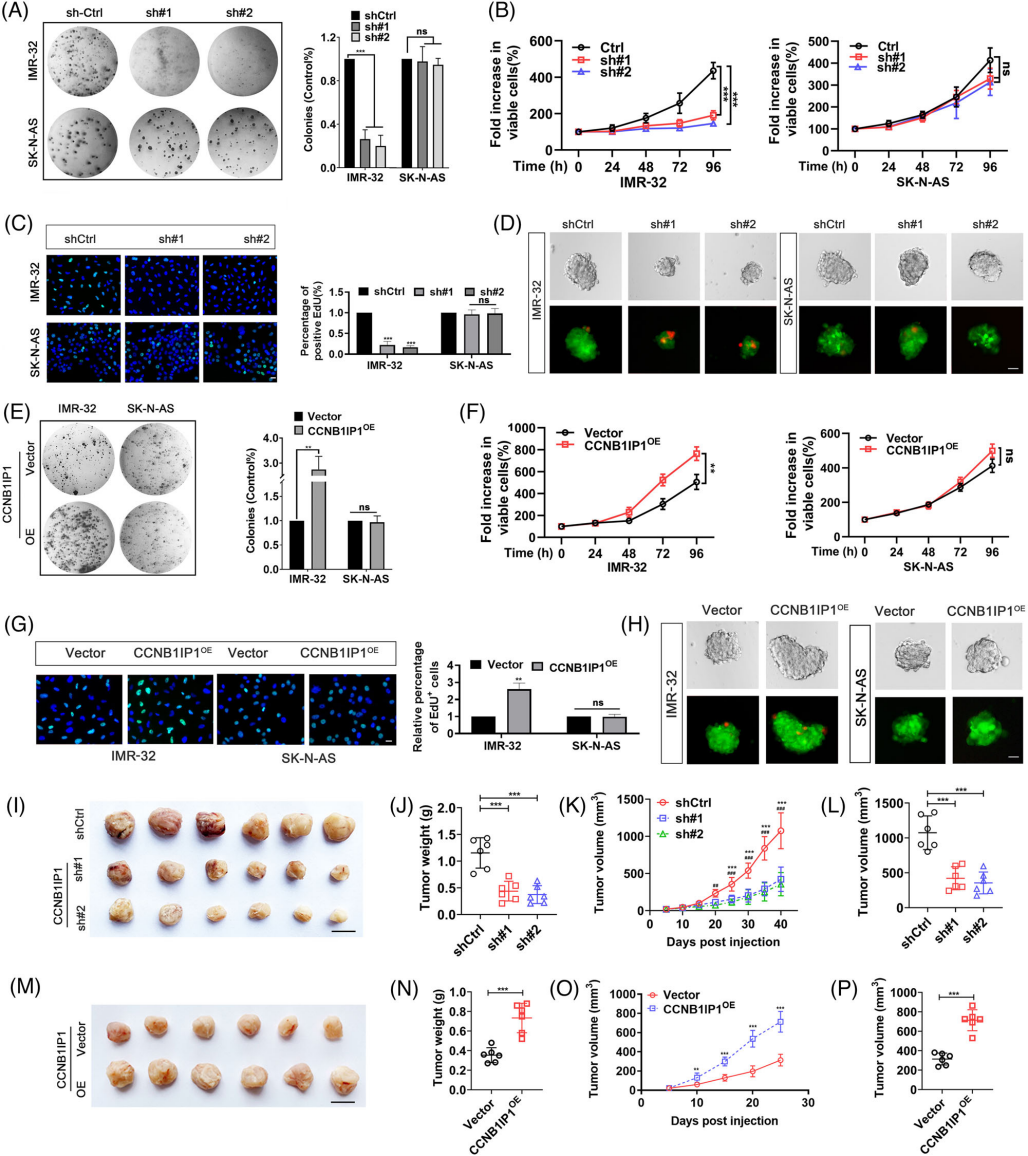
Altering the CCNB1IP1 expression selectively modulates the proliferation and growth of *MYCN*‐AM NB cells. Two shRNAs targeting CCNB1IP1 with different sequences were transfected into NB cells (IMR‐32 and SK‐N‐AS cells) for 48 h. (A) Colony‐formation assay. (B) MTT assay. (C) EdU incorporation assay. (D) Tumour sphere formation assays. Live (green) and dead (red) cells staining. Empty vectors or vectors encoding CCNB1IP1 were transfected into NB cells for 48 h. (E) Colony‐formation assay. (F) MTT assay. (G) EdU incorporation assay. (H) Tumour sphere formation assays. Live (green) and dead (red) cells staining. (I) Image of subcutaneous tumour formation from IMR‐32 cells with or without CCNB1IP1 stably knockdown. Scale bar = 10 mm. (J) Tumor weight. (K) Volume changes of the subcutaneous tumour were measured periodically. (L) Tumour volume at the end of the study. (M) Image of CCNB1IP1 stably expressing IMR‐32 and vector‐IMR‐32‐derived subcutaneous tumours. Scale bar = 10 mm. (N) Tumor weight. (O) The volume changes of subcutaneous tumours were measured periodically. (P) Tumor volume at the study end point. A‐H, data represent the mean ± SD of at least three independent experiments (ns, no significant differences; ^*^ *p* < 0.05; ^**^ *p <* 0.01 and ^***^ *p* < 0.001).

Based on the high expression of CCNB1IP1 in *MYCN*‐AM NB cells, we hypothesized that an excess of CCNB1IP1 facilitated MYCN‐driven tumour‐promoting functions of NB cells. To verify this, we transfected CCNB1IP1‐targeting shRNAs into NB cells with different *MYCN* amplification statuses and examined cell growth and proliferation in vitro. As shown in Figure S4A, two shRNAs targeting CCNB1IP1 exhibited strong silencing efficiency in all six NB cell lines. As shown in Figure 4A and Figure S4B, CCNB1IP1 ablation resulted in a significant suppression of colony formation in *MYCN*‐AM NB cells, whereas little effect was observed in *MYCN*‐NA NB cells. Additionally, the growth of CCNB1IP1‐deficient *MYCN*‐AM NB cells, but not *MYCN*‐NA NB cells, was significantly delayed upon CCNB1IP1 knockdown (Figure 4B). To further clarify the effect of CCNB1IP1 on cell proliferation, an EdU assay was also performed, and as shown in Figure 4C and Figure S4C, a significant reduction in the ratio of EdU‐labeled NB cells was observed in CCNB1IP1‐ablated *MYCN*‐AM NB cells versus controls, but not in *MYCN*‐NA NB cells. As tumour spheroids are more similar to the state of cell survival and more realistic biochemical and physiological responses within tumour tissue than conventional culture methods, tumour sphere formation assays were also performed. As shown in the Figure 4D and Figure S4D knockdown of CCNB1IP1 suppressed the proliferation of spheres and elevated the proportion of dead cells in *MYCN*‐AM cells, whereas it had little effect on *MYCN*‐NA cells. Meanwhile, enhanced cell proliferation was specifically observed in *MYCN*‐AM NB cells upon CCNB1IP1 exogenous expression, as manifested by an increase in the number of colony foci, accelerated cell growth, and an elevated proportion of EdU‐labeled cells and enlargement of tumour spheres, whereas its effect on NA NB cells was inconsistent (Figure 4E–H and Figure S5). To clarify the effect of CCNB1IP1 in NB tumourigenesis, CCNB1IP1‐expressing or deficient NB cells‐derived subcutaneous xenografts mouse models were constructed. Similar to the in vitro results, as shown in Figure 4I–P, depletion of endogenous CCNB1IP1 impaired tumourigenesis whereas exogenous overexpression of CCNB1IP1 facilitated the IMR‐32 cell‐derived tumourigenesis. Altogether, these results suggest that CCNB1IP1 regulates the progression of NB, which appears to be largely dependent on the genetic amplification or expression of MYCN.

### 3.5 CCNB1IP1 stabilizes MYCN protein by suppressing its proteasome‐dependent ubiquitination degradation

**FIGURE 5:**
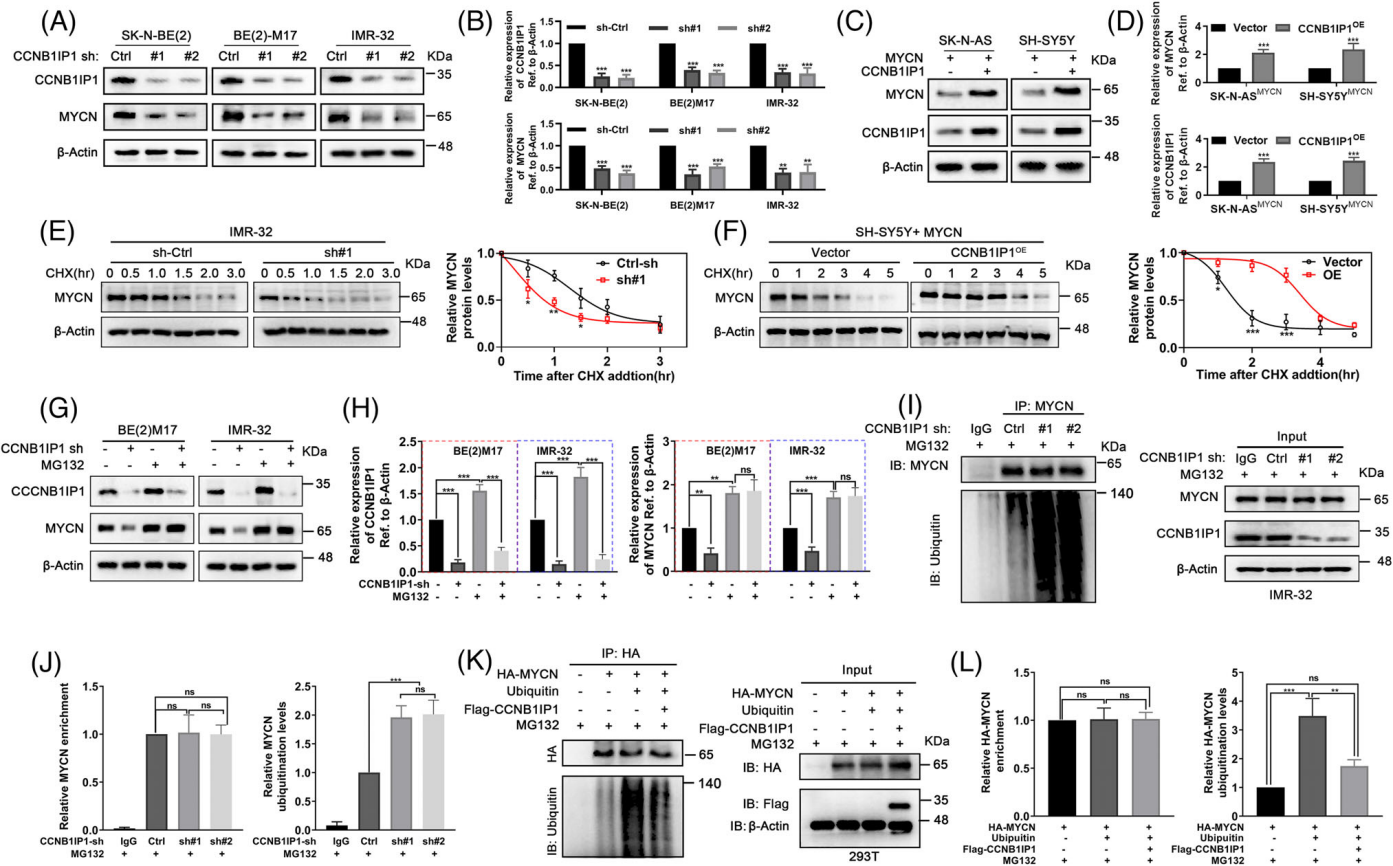
CCNB1I1P1 blocks MYCN protein ubiquitination and degradation. Two shRNAs targeting CCNB1IP1 were transfected into NB cells for 48 h. (A) IB assays were performed to detect indicated protein expression levels. (B) Quantification of **A**. Empty vector or plasmid encoding CCNB1IP1 were transfected into MYCN‐expressing non‐amplified NB cells for 48 h. (C) IB was performed to detect indicated protein expression levels. (D) Quantification of **C**. (E, F) IB assays were performed to assess MYCN stability in NB cells with CCNB1IP1 knockdown or overexpression, which was treated with CHX (10 μg/ml) for the indicated time. (G) The protein expression of CCNB1IP1 and MYCN in CCNB1IP1‐silenced or non‐silenced NB cells stimulated by MG132 (20 μm) was detected by IB assay. (H) Quantification of **G**. (I) Co‐IP assay was performed using the anti‐MYCN antibody in the lysate of IMR‐32 cells with or without CCNB1IP1 knockdown, and then the anti‐ubiquitin antibody was used for detecting MYCN ubiquitination level. (J) Quantification of **I**. (K) HA‐MYCN, Ubiquitin and Flag‐CCNB1IP1 plasmids were transfected alone or together into HEK‐293T, and MYCN ubiquitination level was detected by co‐IP assay. G‐I, MG132 was added to inhibit proteasome‐dependent protein degradation. (L) Quantification of **K**. Data represent the mean ± SD of at least three independent experiments (ns, no significant differences; ^*^ *p* < 0.05; ^**^ *p* < 0.01 and ^***^ *p* < 0.001).

Previous studies have shown that specific factors in the MYCN‐driven gene expression profiling influence the expression or stability of MYCN itself, thus forming a feedback regulatory circuit.^11^, ^12^, ^30^ The above findings confirmed that CCNB1IP1 was highly expressed as an MYCN target gene in *MYCN*‐AM NB cells with a positive effect on their proliferation and tumourigenesis. To investigate whether CCNB1IP1 affects MYCN, we up‐ or down‐regulated CCNB1IP1 in NB cells via transfection of overexpression plasmid or shRNA targeting CCNB1IP1, respectively. As shown in Figure 5A and B and Figure S6A, MYCN protein levels were significantly reduced in CCNB1IP1 knockdown *MYCN*‐AM NB cells, whereas mRNA expression of *MYCN* remained unchanged. Similarly, we observed an upregulation of MYCN protein expression without altering mRNA expression in MYCN‐expressing NA cells upon exogenous overexpression of CCNB1IP1 (Figure 5C and D and Figure S6B). Since MYCN expression at the transcriptional level was not affected by CCNB1IP1, we speculated whether it altered the post‐translational stability of the MYCN protein. Thus, a cycloheximide (CHX) chase assay was performed to evaluate the MYCN protein half‐life. As shown in Figure 5E,F, the degradation of MYCN protein was significantly accelerated upon CCNB1IP1 knockdown, whereas the half‐life of MYCN protein was significantly prolonged with CCNB1IP1 overexpression. Treatment of NB cells with proteasome inhibitor MG132 resulted in increased MYCN protein levels that remained relatively high even under CCNB1IP1 knockdown, suggesting that CCNB1IP1 protects MYCN protein from ubiquitin proteasome‐mediated degradation (Figure 5G). We next performed a co‐IP assay and observed a dramatic increase in the ubiquitination level of MYCN upon CCNB1IP1 knockdown in *MYCN*‐AM NB cells (Figure 5I,J). In a reversal experiment, a significant reduction in MYCN ubiquitination levels was detected in cells exogenously introduced with MYCN after overexpression of CCNB1IP1 (Figure 5K,L). These results suggest that the function of CCNB1IP1 to stabilize MYCN protein is depend on its inhibition of proteasome‐directed ubiquitination modifications.

### 3.6 The proliferation and tumourigenicity of NB mediated by CCNB1IP1 relies on MYCN expression

**FIGURE 6:**
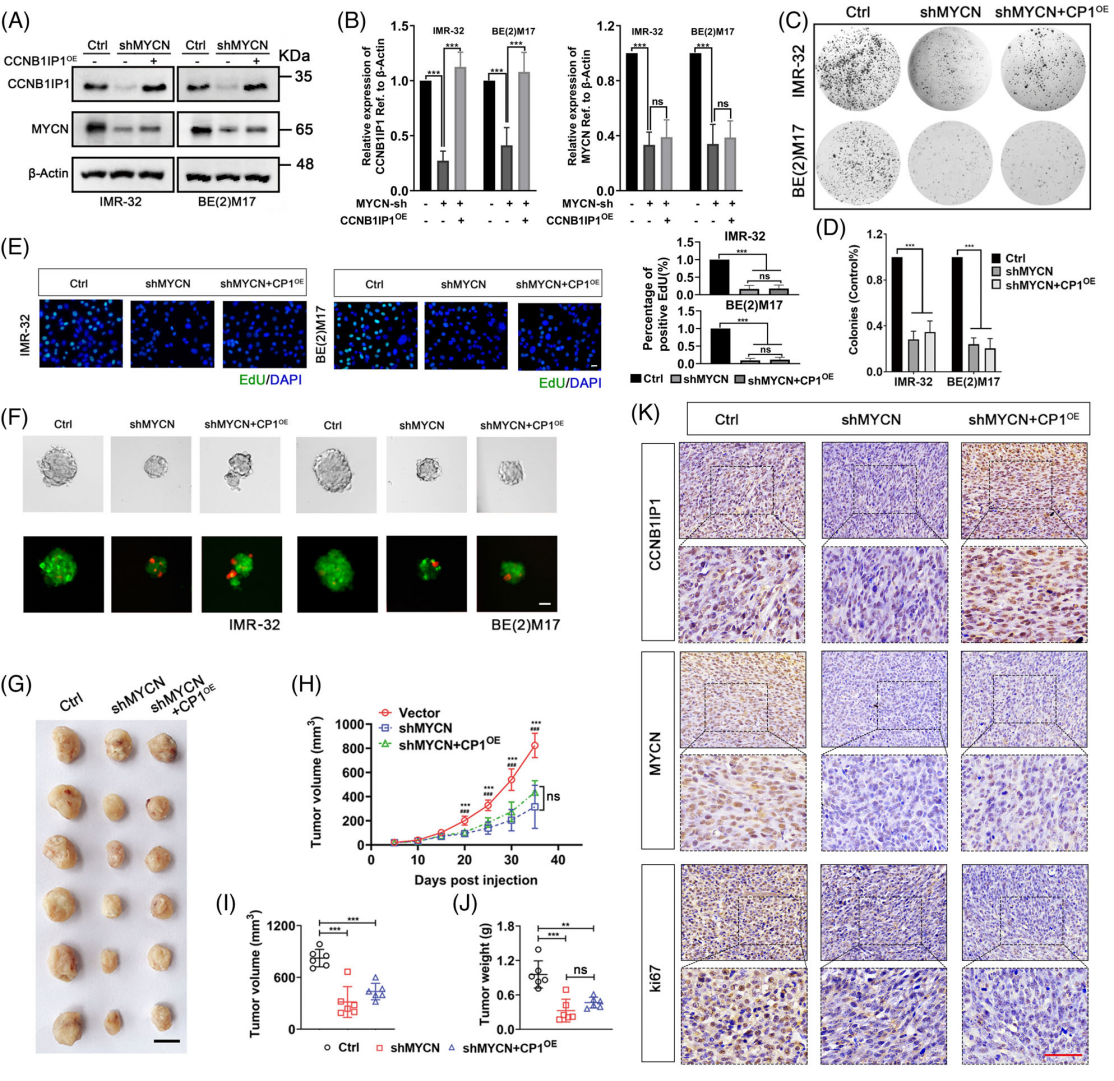
CCNB1IP1 promotes NB cell proliferation and tumour growth in a MYCN‐dependent manner in vitro and in vivo. IMR‐32 and BE(2)M17 cells were transfected with MYCN shRNA alone or together with plasmids encoding CCNB1IP1. (A) IB assay was performed to detect the protein expression of CCNB1IP1 and MYCN. (B) Quantification of **A**. (C) Colony formation assay. (D) Quantification of **C**. (E) EdU staining. (F) Tumor sphere formation. Live (green) and dead (red) cells staining. (G) Image of subcutaneous tumours derived from IMR‐32 cells stably silencing MYCN alone or together with CCNB1IP1 overexpression. Scale bar = 10 mm. (H) The volume changes of tumours were measured periodically. (I) Tumor volume and (J) weight at the study end point. (K) Representative images of IHC staining for CCNB1IP1, MYCN and ki67 in xenografts tissues. Scale bar = 50 μm. A–E, data represent mean ± SD, (ns, no significant differences; ^**^ *p* < 0.01 and ^***^ *p* < 0.001).

Since manipulation of CCNB1IP1 expression exhibited selective regulation of the proliferation and tumourigenic potential of *MYCN*‐AM NB cells, we hypothesized that mRNA or protein expression of MYCN contributes to CCNB1IP1‐mediated oncogenic effects. To investigate this theory, CCNB1IP1 was overexpressed in NB cells with and without MYCN knockdown, and the cells' growth and proliferation were examined by MTT and colony formation assays. As shown in Figure 6A–F, the elevation of CCNB1IP1 by exogenous overexpression plasmid failed to alleviate the inhibition of cell growth, colony formation, EdU incorporation and tumour sphere formation due to MYCN knockdown, indicating that maintenance of MYCN level is critical for CCNB1IP1 to foster cell growth and proliferation. To further clarify the effect of CCNB1IP1 on *MYCN*‐AM NB cell tumour growth, we constructed Ctrl‐, shMYCN‐ and shMYCN+CCNB1IP‐IMR‐32 cells‐derived subcutaneous xenograft tumour models. As shown in Figure 6G–J, xenografts in the shMYCN group were significantly reduced in size, weight and growth rate compared with the Ctrl group, whereas tumour suppression was not alleviated in the shMYCN+CCNB1IP1 group, even with the rescue of CCNB1IP1 expression. In addition, IHC results confirmed that CCNB1IP1 immunostaining was attenuated in the MYCN knockdown group and rescued in the CCNB1IP1 overexpression group in tumour tissues (Figure 6K). However, consistent with the tumour growth phenotype, restoration of CCNB1IP1 expression did not effectively reverse tumour proliferation suppression caused by MYCN silencing, as evidenced by the unchanged Ki67 staining intensity in the shMYCN+CCNB1IP1^OE^ group versus the shMYCN group (Figure 6K). The above results suggest that the advantageous effect of CCNB1IP1 on NB cell proliferation and tumour growth depends on the high expression level of MYCN.

### 3.7 Stabilization of MYCN protein mediated by CCNB1IP1 is associated with disruption of FBXW7‐mediated ubiquitination

**FIGURE 7:**
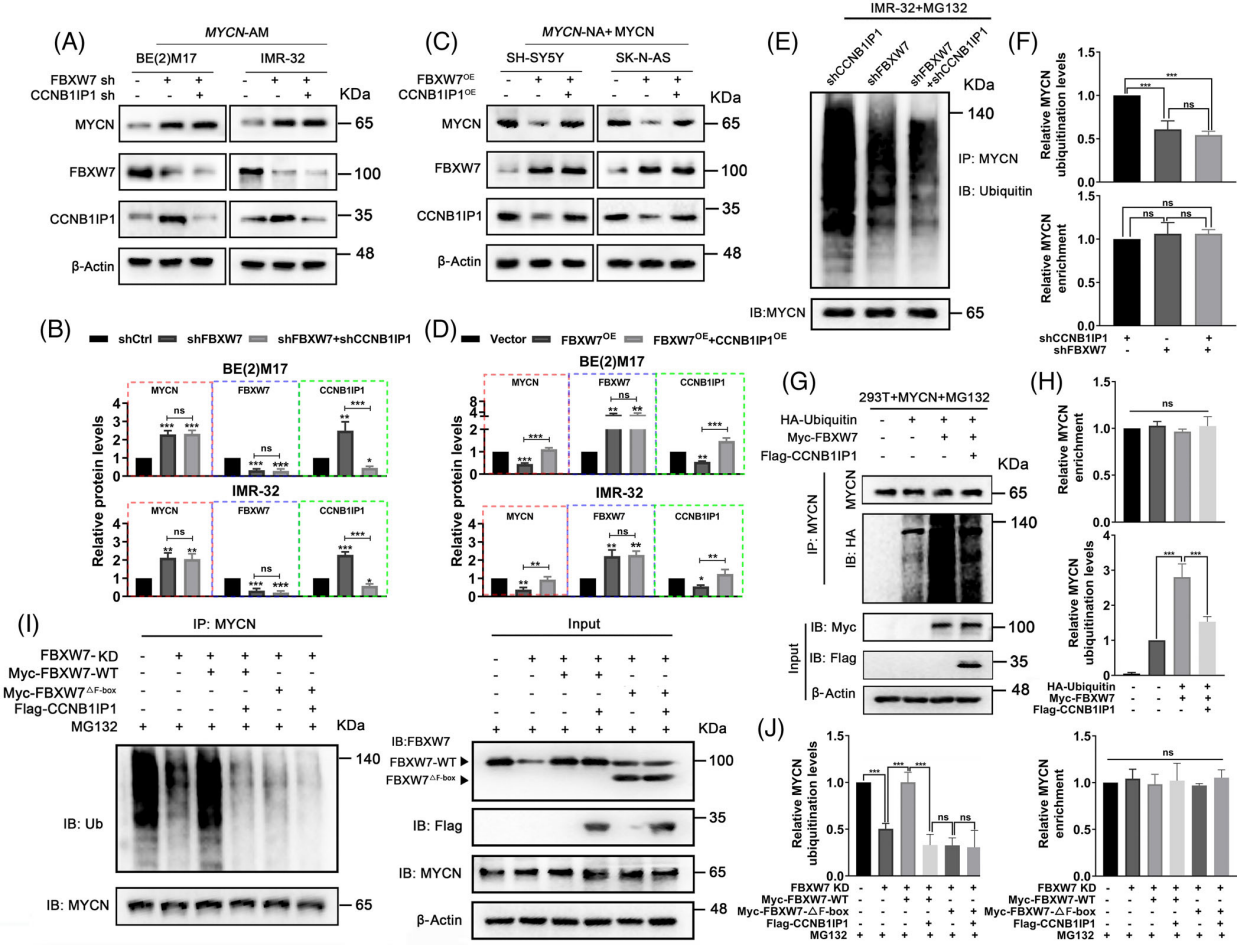
CCNB1IP1 stabilizes MYCN protein by disrupting FBXW7‐mediated ubiquitination. IB analysis of MYCN, FBXW7 and CCNB1IP1 protein expression. (A) BE(2)M17 and IMR‐32 cells infected with shFBXW7 alone or together with shCCNB1IP1. (B) Quantification of **A**. (C) SH‐SY5Y and SK‐N‐AS cells overexpressed FBXW7 alone or together with CCNB1IP1. (D) Quantification of **C**. (E) In vivo ubiquitination assay of MYCN in IMR‐32 cells with knockdown of FBXW7 and CCNB1IP1 alone or together. (F) Quantification of **E**. In vivo ubiquitination assay of MYCN. (G) MYCN‐expressing HEK293T cells infected with plasmids encoding HA‐Ubiquitin, Myc‐FBXW7 and Flag‐CCNB1IP1 alone or together. (H) Quantification of **G**. (I) IMR‐32 cells knockdown of FBXW7 infected with plasmids encoding Myc‐FBXW7‐WT, Myc‐FBXW7^△F‐box^ and Flag‐CCNB1IP1 alone or together. (J) Quantification of **I**. MG132 was added to inhibit proteasome‐dependent protein degradation. Data represent mean ± SD, (ns, no significant differences; ^**^ *p* < 0.01 and ^***^ *p* < 0.001).

As a short‐lived protein, MYCN stability tends to be tightly modulated by multiple intracellular proteasomal ubiquitin‐dependent pathways involved in several well‐studied ubiquitin ligases or deubiquitinating enzymes, such as Trim32, FBXW7, Huwe1, USP3 and USP5.^10^, ^31^, ^32^, ^33^, ^34^ Among them, Trim32, FBXW7 and Huwe1 as ubiquitin ligase exert a negative regulatory effect in MYCN protein stability, whereas USP3 and USP5 as deubiquitinating enzymes elicited the opposite effect. Next, we examined which signalling pathways were involved in the CCNB1IP1‐mediated stabilization of MYCN via post‐transcriptional regulation. As shown in Figure S7, ablation of CCNB1IP1 partially prevented the upregulation of MYCN protein level in Trim32 or Huwe1 knockdown cells, and overexpression of CCNB1IP1 differentially attenuated the downregulation of MYCN protein level caused by USP3 or USP5 silencing. However, no significant effect was observed in FBXW7 knockdown cells (Figure 7A,B). As shown in Figure 7C,D, MYCN expression was downregulated following the overexpression of FBXW7. And the expression of CCNB1IP1 was also reduced, which may be the result of the reduction of MYCN. Further exogenous expression of CCNB1IP1 was able to rescue the FBXW7‐mediated MYCN downregulation. Additionally, CCNB1IP1 expression did not noticeably alter the protein expression level of FBXW7 both in *MYCN*‐AM and NA NB cells. Compared to CCNB1IP1 knockdown alone, MYCN protein ubiquitination level was significantly decreased in IMR‐32 cells after FBXW7 knockdown, but no further restorative elevation was observed after co‐knockdown of CCNB1IP1 (Figure 7E,F). In contrast, a significant inhibitory effect of elevated CCNB1IP1 expression on FBXW7‐mediated MYCN ubiquitination was observed in HEK293T cells that exogenously expressed MYCN (Figure 7G,H). This seems to indicate that the degradation of MYCN modulated by CCNB1IP1 may involve a functional blockade of FBXW7. To confirm whether the function of FBXW7 is required for CCNB1IP1‐regulated MYCN ubiquitination modification, we re‐expressed FBXW7 WT or function‐deficient mutant F‐box deletion mutant (FBXW7^△F‐box^) in FBXW7‐knockdown cells. As shown in Figure 7I,J, overexpression of CCNB1IP1 significantly counteracted WT FBXW7‐induced MYCN ubiquitination, whereas it failed to cause further down‐regulation of MYCN ubiquitination upon expression of FBXW7 with an F‐box deletion mutation. Through these experiments, we observed that increasing CCNB1IP1 expression improved MYCN protein stability when FBXW7 was expressed, while in the absence of FBXW7 expression or functional inactivation, MYCN protein expression became more stable and was no longer regulated by CCNB1IP1. These findings suggest that CCNB1IP1 stabilizes MYCN protein mainly as a result of counteracting FBXW7‐mediated ubiquitination degradation of MYCN.

### 3.8 CCNB1IP1 maintains MYCN protein stability by competing with FBXW7 for MYCN binding

**FIGURE 8:**
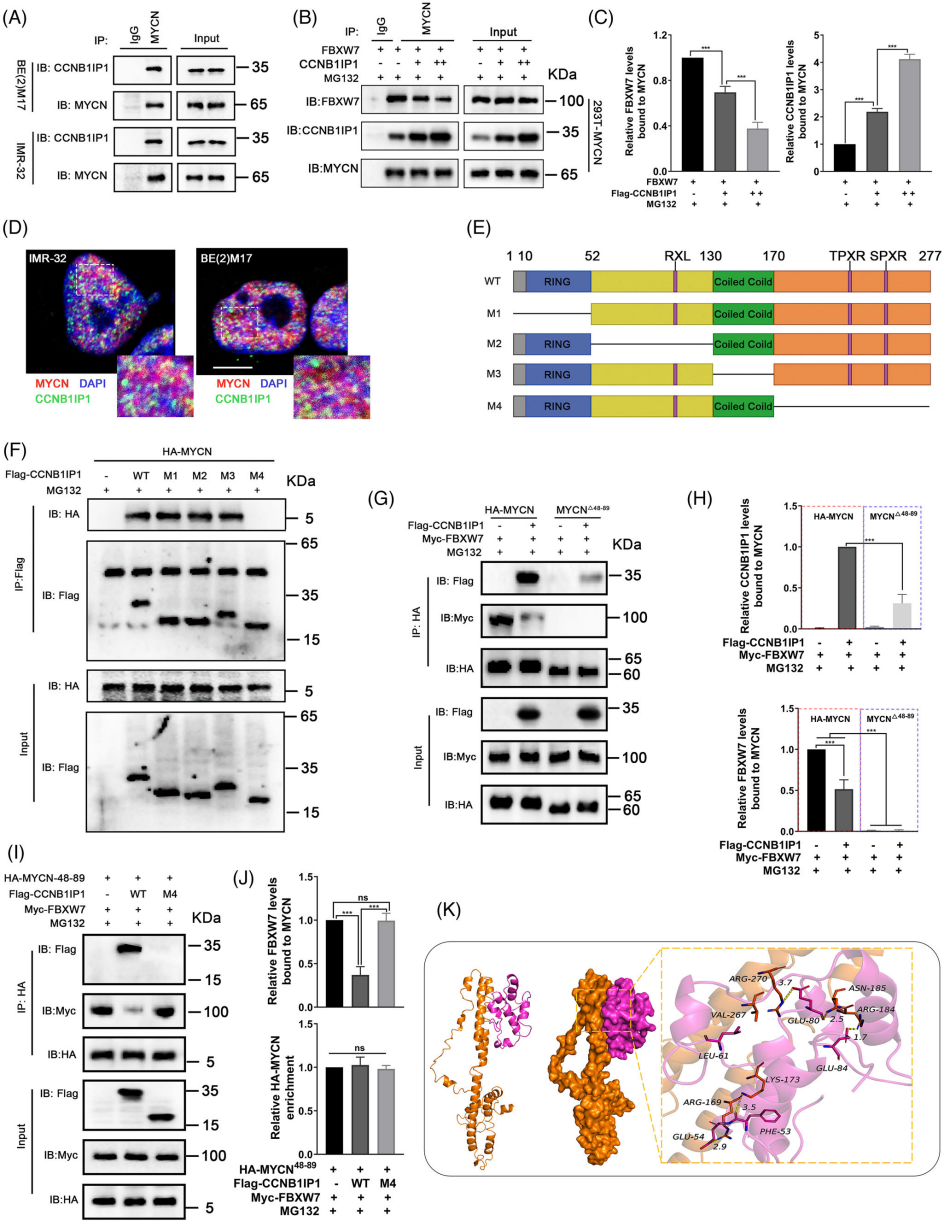
CCNB1IP1 stabilizes MYCN protein by competing with FBXW7 for MYCN binding. (A) Endogenous interaction between CCNB1IP1 and MYCN. Co‐IP assay was performed using anti‐MYCN antibody and IgG was used as a negative control. (B) Interference of exogenously expressed CCNB1IP1 with the FBXW7‐MYCN interaction was detected by co‐IP assay. (C) Quantification of **B**. (D) The localization of MYCN and CCNB1IP1 was detected using the IF method in IMR‐32 and BE(2) M17 cells. Co‐localization analysis was performed using ImageJ and the plugin JACoP. Manders' Coefficients (M1: fraction of MYCN overlapping CCNB1IP1, M2: fraction of CCNB1IP1 overlapping MYCN) for IMR‐32: M1 = 0.67 ± 0.07, M2 = 0.82 ± 0.06 and for BE(2)M17: M1 = 0.63 ± 0.07, M2 = 0.93 ± 0.05.) Scale bar = 5 μm. (E) Schematic depiction of CCNB1IP1 WT and truncated mutants. (F) The interaction between MYCN and CCNB1IP1 WT or truncated mutants was determined in HEK293T cells transfected separately with Flag‐CCNB1IP1 WT and different truncated mutants along with HA‐MYCN. Co‐IP assay to analyze the competitive binding of CCNB1IP1 to MYCN with FBXW7. (G) HEK293T cells expressing HA‐tagged MYCN WT or MYCN^△48‐89^ mutant were transfected with plasmids encoding Myc‐FBXW7 alone or together with Flag‐CCNB1IP1. (H) Quantification of **G**. (I) HEK293T cells expressing Myc‐FBXW7 were transfected with plasmids encoding HA‐MYCN WT or MYCN^48‐89^ mutant alone or together with Flag‐CCNB1IP1. (J) Quantification of **B**. (K) Detailed binding pattern of CCNB1IP1 to MYCN. The backbone of protein was rendered in a tube and coloured. CCNB1IP1 (down) and MYCN (up) proteins are rendered by the surface. The yellow dashes represent hydrogen bonds.

It has been shown that the recognition and binding of MYCN by FBXW7 determines the degradation of MYCN.^20^, ^35^ As shown in Figure 8A, co‐IP assay showed the endogenous interaction of CCNB1IP1 with MYCN in BE(2)M17 and IMR‐32 cells. Thus, we hypothesized whether CCNB1IP1 might affect the interaction of FBXW7 with MYCN. As shown in Figure 8B and C and Figure S8, overexpression of CCNB1IP1 blocked whereas knockdown of CCNB1IP1 increased the binding of FBXW7 to MYCN in a CCNB1IP1 expression‐dependent manner. To observe the intracellular localization of MYCN and CCNB1IP1 expression, we conducted IF experiments as well as co‐localization analysis. As described in Figure 8D, MYCN and CCNB1IP1 were abundantly co‐localized in the nucleus of NB cells, which further corroborated their interaction. To further investigate the disruption of FBXW7‐MYCN interaction by CCNB1IP1, a co‐IP assay was performed in HEK293T cells transfected with the constructed plasmids encoding HA‐MYCN and Flag‐CCNB1IP1 WT or truncated mutants. As shown in Figure 8E,F, HA‐MYCN evidently interacted with Flag‐CCNB1IP1 WT and M1‐3 truncation mutants, but not with Flag‐CCNB1IP1‐M4 truncation mutant, suggesting that the C‐terminal domain of CCNB1IP1 is a critical binding site for MYCN. It has been previously shown that MYCN AA 48 to 89 determined the interaction with FBXW7.^20^ We next investigated the influence of MYCN AA 48 to 89 for the binding of MYCN between CCNB1IP1 and FBXW7. As shown in Figure 8G,H, Flag‐CCNB1IP1 significantly restrained the co‐binding of Myc‐tagged FBXW7 with HA‐MYCN. However, the HA‐MYCN mutant with deletion of AA 48 to 89 (HA‐MYCN^△48‐89^) greatly impaired the capacity to bind both Myc‐FBXW7 and Flag‐CCNB1IP1. And to fully characterize their interaction, the constructed expression vectors encoding AA 48 to 89 of HA‐MYCN (HA‐MYCN^48‐89^), Flag‐CCNB1IP1 WT, M4 and Myc‐FBXW7 were either separately or co‐transfected into HEK293T cells. As shown in Figure 8I,J, both Flag‐CCNB1IP1 WT and Myc‐FBXW7 were able to interact with HA‐tagged MYCN^48–89^, whereas the Flag‐CCNB1IP1 M4 mutant showed no physical binding to MYCN^48‐89^. In addition, Flag‐CCNB1IP1 WT but not the M4 mutant was able to impair the FBXW7‐MYCN^48‐89^ interaction. Based on these results we also simulated the interaction between CCNB1IP1 and MYCN by computer‐assisted protein‐protein docking. As shown in Figure 8K, the protein binding of CCNB1IP1 to MYCN was scored as −225.12 kcal/mol. The multiple interactions formed by residues in contact between CCNB1IP1 and MYCN proteins, such as hydrogen bonding and hydrophobic interactions, may effectively enhance the stability of the protein complexes. Therefore, we conclude that CCNB1IP1 is able to bind the identical MYCN amino acid region to FBXW7, and also reveal that the competitive binding effect of CCNB1IP1 is responsible for the escape of MYCN from FBXW7‐mediated ubiquitination degradation.

### 3.9 CCNB1IP1 stabilizes MYCN and promotes oncogenicity in a C‐terminal domain‐dependent manner

**FIGURE 9:**
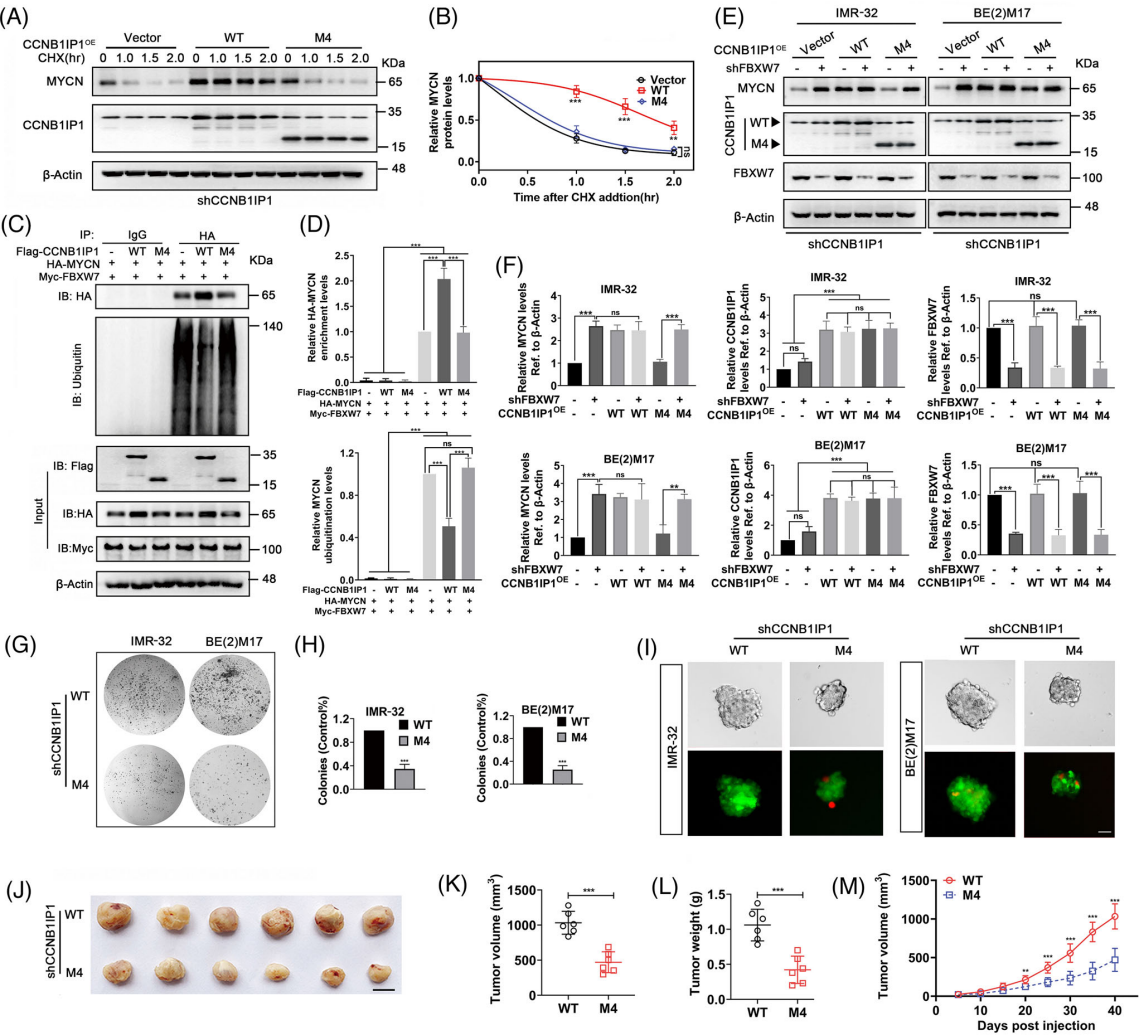
The C‐terminal domain of CCNB1IP1 is essential for MYCN stabilization, and promotion of tumour growth. (A) Protein half‐life analysis of MYCN in shCCNB1IP1‐IMR‐32 cells with re‐expression of CCNB1IP1 WT or M4 mutant plasmids. An empty vector was used as a negative control. Cells were treated with CHX (10 μg/ml) and lysates were harvested for IB analysis. (B) Quantification of **A**. (C) Ubiquitination of MYCN was detected in HEK293T cells transfected with HA‐MYCN alone or together with Myc‐FBXW7, Flag‐CCNB1IP1 WT or M4 mutant. CCNB1IP1 WT or M4 mutant was re‐expressed alone or co‐transfected with FBXW7‐shRNA in CCNB1IP1‐depleted NB cells. (D) Quantification of **C**. (E) IB assay was performed to detect the expression of specific proteins. (F) Quantification of **E**. The shCCNB1IP1‐IMR‐32 and ‐BE(2) M17 cells were transfected with CCNB1IP1 WT or M4 mutant plasmids. (G) Colony formation assays. (H) Quantification of **G**. (I) Tumor sphere formation. Live (green) and dead (red) cell staining were performed. CCNB1IP1‐depleted IMR‐32 cells stably re‐expressing CCNB1IP1 WT or M4 mutant were then subcutaneously inoculated into nude mice to construct xenograft tumour models. (J) Images of the xenografts. Scale bar = 10 mm. (K) Tumor volume (L) and weight at the end point. (M) Volume changes of subcutaneous tumours were measured periodically. A–I, data represent the mean ± SD of at least three independent experiments (ns, no significant differences; ^**^ *p* < 0.01 and ^***^ *p* < 0.001).

The above results suggest that the C‐terminal domain of CCNB1IP1 is essential for the competitive binding of MYCN with FBXW7. To further clarify whether this region is required for MYCN stability, we conducted a knockdown of CCNB1IP1 in IMR‐32 cells and re‐expressed the CCNB1IP1 WT or M4 mutant. MYCN protein degradation was detected by CHX‐chase assay. As shown in Figure 9A,B, overexpression of CCNB1IP1 WT, but not M4 mutant, significantly rescued the reduction in MYCN half‐life caused by CCNB1IP1 knockdown. Interestingly, overexpression of CCNB1IP1 WT was able to suppress the ubiquitination of MYCN and stabilize its protein level, whereas this did not occur with the M4 mutant of CCNB1IP1 (Figure 9C,D). To verify whether this stabilizing effect of CCNB1IP1 is associated with its interference with FBXW7, we re‐expressed CCNB1IP1 WT and M4 mutant in CCNB1IP1 knockdown NB cells with or without FBXW7 silencing. As shown in Figure 9E,F, silencing FBXW7 significantly upregulated MYCN protein levels in CCNB1IP1 knockdown cells, whereas there was no obvious effect on the restoration of MYCN protein in cells re‐expressing CCNB1IP1 WT. However, re‐expressing the M4 mutant failed to reverse the down‐regulation of MYCN protein and failed to further interfere with the regulation of MYCN expression by FBXW7. Given that CCNB1IP1 regulated the protein stability of MYCN and is involved in MYCN‐AM NB cell proliferation and oncogenesis, we speculated whether the function of CCNB1IP1 in stabilizing MYCN protein determines its oncogenicity. As shown in Figure 9G–I, the colony and tumour sphere formation ability of MYCN‐AM NB cells re‐expressed M4 mutant was significantly diminished compared to those transfected with CCNB1IP1 WT. To this end, in vivo experiments were also conducted with an IMR‐32 cells‐derived xenograft mouse model. As shown in Figure 9J–M, shCCNB1IP1‐IMR‐32 cells re‐expressing the CCNB1IP1 M4 mutant showed significant tumour growth inhibition compared to cells re‐expressing CCNB1IP1 WT. The data suggest that the C‐terminal domain of CCNB1IP1‐mediated deubiquitination and stabilization of MYCN are essential for tumourigenesis in NB.

## 4 DISCUSSION

In view of the “undruggability” of MYCN, indirect targeting strategies through manipulating interactive molecules associated with MYCN have been rapidly discovered and developed over the years, which has become a research hotspot in the NB field.^8^ Studies have confirmed that the growth of *MYCN*‐AM NB cells is highly dependent on certain genes downstream of MYCN, which may be unnecessary for *MYCN*‐NA NB cells.^9^, ^10^ Manipulation of these genes would selectively suppress the malignant biological phenotype of MYCN‐AM NB, which exemplifies the promising potential of this strategy in drug development and clinical applications.^35^, ^36^, ^37^ Therefore, it is of profound relevance to search for potential regulatory factors and elaborate new strategies based on the specific activation pathways of *MYCN* amplification from the perspective of “selective inhibition”. For this, we identified 15 shared differential genes based on transcriptomic differences between *MYCN*‐AM and NA NB tissues and cell lines in multiple datasets, which also contained several potential targets for NB that have been and are being investigated in NB, such as ornithine decarboxylase‐1 (ODC1), phosphoglycerate dehydrogenase (PHGDH) and Aurora kinase B (AURKB).^38^, ^39^, ^40^ Upon further screening based on the correlation analysis of MYCN expression, we noted that CCNB1IP1 showed a high positive correlation with MYCN expression in all datasets, and thus CCNB1IP1 was considered an objective for further study.

Presently, studies on the expression and biological functions of CCNB1IP1 in tumours, especially in NB, are still relatively unexplored. It was reported that silencing CCNB1IP1 in gastric cancer cells U2OS and breast cancer cells MCF‐7 promoted cell metastasis and invasion, but suppressed cell proliferation and growth, suggesting that the extent or nature of CCNB1IP1 being required in different biological behaviours of tumour cells may not be consistent.^18^ In this manner, we examined the role of CCNB1IP1 in NB from limited studies. We noted a high level of upregulation of CCNB1IP1 in Cre‐conditional MYCN‐driven NB mouse model tumour tissue compared to normal adrenal tissue in a set of transcriptomic data reported by K Althoff et al.^41^ Furthermore, transcriptomic data from Orli Yogev et al. in TH‐MYCN mice showed that CCNB1IP1 was significantly upregulated in NB tissues in a spontaneous mouse model of cyclophosphamide resistance compared to tissues without chemotherapy.^19^

Although the role of CCNB1IP1 was not further investigated, the results partially implicated a potential role of CCNB1IP1 in tumourigenesis as well as chemoresistance in NB. We considered CCNB1IP1 as a novel MYCN‐interacting molecule in NB tumourigenicity that is worthy of attention and exploration. Of course, differential genes other than CCNB1IP1 are also capable of facilitating the MYCN‐driven malignant development of NB through proliferation, metastasis, metabolism and other biological behaviours. A classical case includes the Aurora kinase inhibitor CCT137690, which inhibits MYCN protein expression leading to chromosomal mislocalization and apoptosis, demonstrating potent anti‐tumour effects in *MYCN*‐AM NB cell lines as well as in primary tumour models.^42^ Furthermore, some investigators found that the inhibition of ODC1, a direct target of MYCN, inhibited neuroblastoma cell proliferation, prevented spontaneous tumourigenesis in mice, and enhanced the antitumour efficacy of conventional cytotoxicity.^38^ The drawback of this study is that only CCNB1IP1 alone was selected as a candidate to investigate. Changes in the survival status of *MYCN*‐AM NB cells may be more informative for identifying key targets if the shared differential genes that were screened could be interfered with individually or in different combinations.

In recent years, many studies on the mechanism and application of direct targeting of MYCN downstream targets have emerged. A few mechanism‐based inhibitors with superior antitumour effects have been developed, with some of these inhibitors having entered clinical studies. For example, AURKA as a downstream target of MYCN is also involved in regulating the stability of MYCN protein.^10^ MLN8237 (alisertib), an inhibitor capable of distorting the AURKA conformation, has achieved favourable responses in phase I and phase II clinical trials in combination with irinotecan and temozolomide for the treatment of relapsed or refractory neuroblastoma, which may be a potentially more favourable application of this inhibitor in combination with chemotherapeutic drug therapies.^43^, ^44^ Similarly, a newly identified MYCN cofactor with similar effects is PA2G4, although it is still in preclinical studies.^12^ These studies indicate that drug development based on identified key molecules that regulate MYCN or are regulated by MYCN can definitely be a promising approach. We note that all of these attractive potential targets do not simply perform biological functions as target genes of MYCN, but are more likely to generate powerful oncogenic effects in their interaction with MYCN. Similarly, in the present study, we found that CCNB1IP1 not only acts as a downstream target gene of MYCN, but more importantly, it is involved in regulating the protein stability of MYCN, which provides substantial support for the tumourigenicity of MYCN‐driven NB cells. Moreover, disruption of the interaction between CCNB1IP1 and MYCN led to suppressed proliferation and growth of *MYCN*‐AM NB cells. Interfering with the post‐transcriptional stability or activity of MYCN is relatively straightforward and the development of more effective and feasible options to target MYCN is receiving increased attention. Therefore, we propose that CCNB1IP1 is an important candidate target worth investigating. The future development of inhibitors targeting MYCN/CCNB1IP1 interaction will likely benefit MYCN‐AM NB patients, a high‐risk group.

The strategies for targeting MYCN are diverse. Direct disruption of the regulatory function of MYCN on target genes also represents another promising means. It is well known that MYCN forms heterodimers with MAX that bind to E‐box regulatory DNA elements, thereby controlling the transcription of a large set of genes and their proteins that favour the malignant phenotype of cells.^45^ In NB, attempts have been made to target MYCN‐MAX dimerization or inhibit MYCN‐MAX binding to DNA to block and arrest tumour development, and this has been a very long‐standing and relatively attractive perspective. Although there are few studies based on this mechanism in NB, well‐developed small molecule inhibitors have been developed, such as compound 10058‐F4^46^ and MYC‐MAX dimerization inhibitor OmoMYC.^47^ In terms of NB, however, most studies have been conducted in cells in vitro and the effects of tumour treatment in vivo are still largely unknown. Although inhibition of CCNB1IP1 alone could not fundamentally eliminate tumour cells, it greatly delayed and inhibited tumour proliferation and growth in vitro and in vivo. In addition, we note that numerous previous studies have shown that either inhibition of the downstream target genes of MYCN alone or its own regulatory function failed to completely inhibit tumour growth. And even under some combination treatments, there will always be cells that can circumvent the inhibitory signals and survive. Indeed, this is one of the reasons why tumour cells with extremely complex regulatory networks are difficult to deal with. Therefore, it seems difficult to conclusively determine which intervention pathway is an end‐all means for tumour suppression. Importantly, there is still a need to raise awareness and consider whether tumour cells that escape growth inhibition may rely on other signalling pathways for survival. Future targeted combination interventions with CCNB1IP1 and other related survival escape targets potentially could lead to a new horizon for better treatment.

According to our data, the regulation of MYCN protein ability by CCNB1IP1 is mainly associated with E3 ubiquitin ligase FBXW7 and is not dependent on other partner molecules interacting with MYCN (Trim32, Huwe1, USP3 and USP5) (Figure 7 and Figure S7).^10^, ^31^, ^32^, ^33^, ^34^ In truncated mutation experiments, we found that CCNB1IP1 bound tightly to the reciprocal region of FBXW7 on MYCN AA 48−89 in a C‐terminal domain‐dependent manner (Figure 8). Interestingly, MYCN with deletion mutations in this region lost its ability to bind FBXW7, and binding to CCNB1IP1 was also significantly abolished. This indicates that the FBXW7 binding segment is contained at least within the region where MYCN interacts with CCNB1IP1. In contrast, the MYCN AA 48−89 regions are not required for the binding of other MYCN cofactors (Trim32, Huwe1, USP3 and USP5). Therefore, CCNB1IP1 would not disturb their binding to MYCN or further affect the stability of MYCN protein, which presumably corroborates with the results shown in Figure 7 and Figure S7.

Overall, our data supported the potential of CCNB1IP1 as a candidate for targeting MYCN. Our results, along with other reports in the literature, adequately indicate that the destabilization of MYCN is going to be an overwhelming breakthrough in attacking *MYCN* NA NB. However, there remain certain limitations and pending issues to be addressed in our study. Although the destabilization of MYCN has great potential as an emerging therapeutic strategy, it also faces various obstacles, such as specificity and clinical translation challenges. We found that although CCNB1IP1 was overexpressed in *MYCN*‐AM NB, it was also detectable in NA samples and cells, so perhaps its expression in NB is not specific. Of course, CCNB1IP1 is expressed in cells with its normal regulatory functions, such as regulating CyclinB1 expression to manipulate cell cycle progression.^48^ We speculated that the overexpression of CCNB1IP1 may exercise a specific regulatory function in cells with high MYCN expression, and the precise regulatory mechanism remains to be further clarified. At least in the present study, we concluded that CCNB1IP1 in collaboration with MYCN did produce a potent promotion effect on NB cell proliferation and growth, whereas manipulating CCNB1IP1 alone did not work when MYCN was absent or lowly expressed. Furthermore, we have currently focused only on the effects of CCNB1IP1 on NB cell proliferation and xenograft growth in vivo, whereas other critical biological phenotypes in tumourigenesis, drug resistance, metastasis, invasion, cell death, differentiation and autophagy remain unexplored.^49^, ^50^ It would be attractive and meaningful to comprehensively investigate the biological function of CCNB1IP1 in NB and to clarify whether this is entirely dependent on MYCN expression. The functional study of CCNB1IP1 in NB may directly reflect the effectiveness and clinical translation potential of targeting CCNB1IP1 for the treatment of *MYCN*‐AM patients.

Although no definitive inhibitor of CCNB1IP1 has been developed, our study indicates that targeting CCNB1IP1 may be a new option for the future treatment of *MYCN*‐AM NB. Screening and validation of small molecule compounds or agents with known clinical indications that specifically disrupt the interaction of CCNB1IP1 with MYCN as candidate inhibitors can be further explored through preclinical testing and subsequent clinical applications of *MYCN*‐AM NB. Although targeting CCNB1IP1 is still far from clinical application, it remains undeniable that NB cell growth is impaired by disrupting the CCNB1IP1‐MYCN interaction. Ideally, more effective drug combination regimens could be developed based on this idea to provide more effective and specific treatments for improving the prognosis and survival of high‐risk NB patients.

## 5 CONCLUSION

**FIGURE 10:**
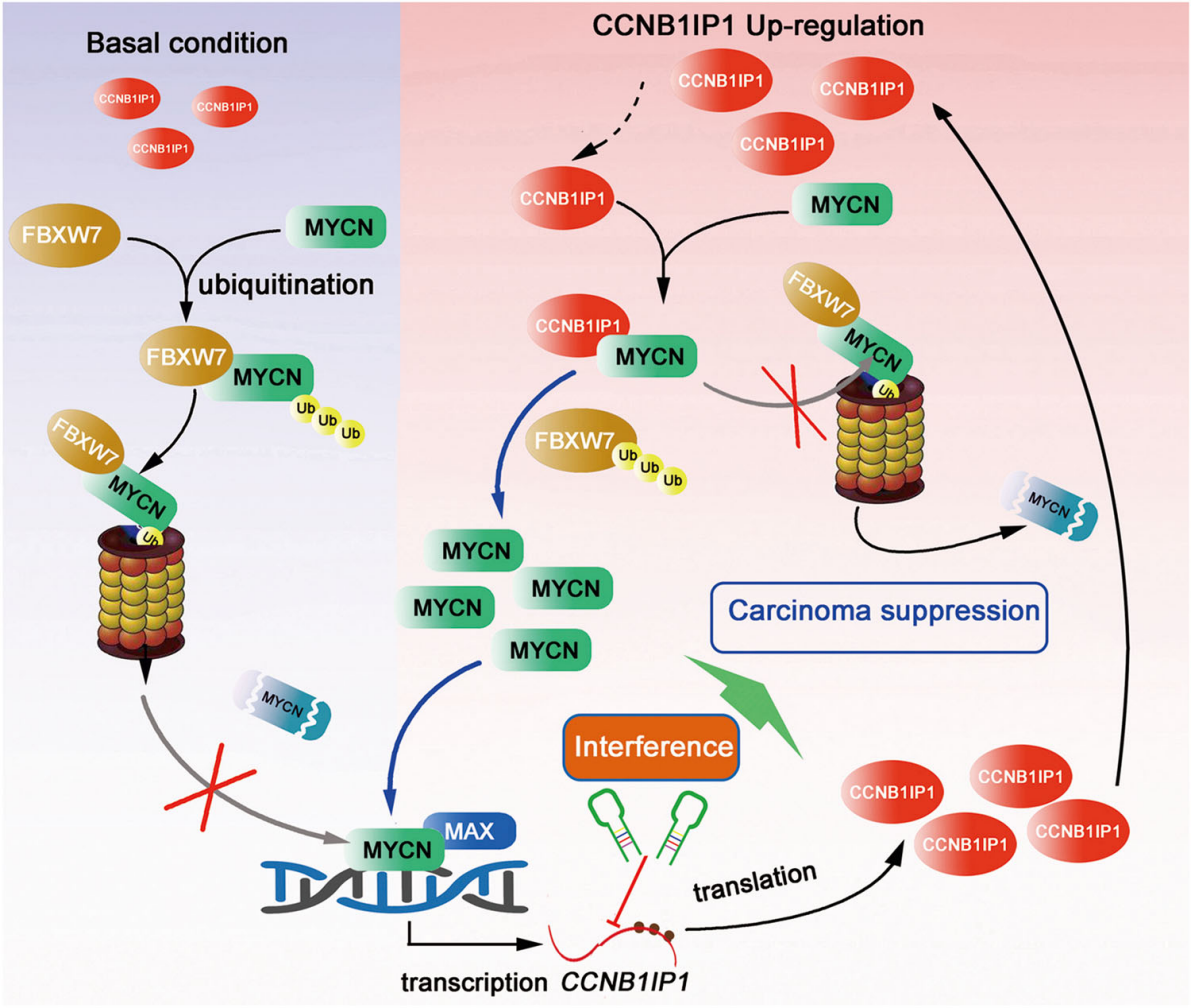
Proposed model of the disruption of FBXW7‐mediated MYCN ubiquitination degradation by CCNB1IP1 in NB cells. In MYCN‐amplified NB, MYCN directly induces the transcription of CCNB1IP1. CCNB1IP1 stabilizes MYCN protein by inhibiting FBXW7‐mediated degradation of MYCN ubiquitination in a competitive binding manner. Interfering with CCNB1IP1 reverses this effect and suppresses MYCN‐driven tumourigenicity in NB.

In this study, we identified CCNB1IP1 as a novel cofactor that stabilizes MYCN and acts synergistically with MYCN to enhance the proliferation and tumourigenicity of NB cells. Mechanistically, CCNB1IP1 blocked MYCN degradation in a C‐terminal‐dependent manner through the competitive binding of MYCN to FBXW7, which further diversified the regulatory mechanism of MYCN protein stabilization. Dissecting the role and therapeutic potential of CCNB1IP1 in MYCN‐driven carcinogenesis will enable a better understanding and provide a rational explanation for its use as a new therapeutic strategy and target for NB (Figure 10).

## CONFLICT OF INTEREST STATEMENT

The authors declare no conflict of interest.

## FUNDING INFORMATION

This work was supported by grants from the Henan Medical Science and Technology Project (LHGJ20210629, LHGJ20190958 and LHGJ20190889), and the Scientific and Technological Projects of Henan province (222102310026), the Natural Science Foundation of China (No. 82172558).

## Supporting information

Figures S1–S8

Tables S1–S5

## Data Availability

The datasets used and/or analyzed in the current study are available from the corresponding author upon reasonable request.
