# Supplementary material for: CCNB1IP1 prevents ubiquitination‐mediated destabilization of MYCN and potentiates tumourigenesis of MYCN‐amplificated neuroblastoma
Source: Clin Transl Med. 2023 Jul 17;13(7):e1328. doi: 10.1002/ctm2.1328 (PMC10352605; doi:10.1002/ctm2.1328)
Supplement: Supplementary file 1 — Figures S1–S8 [file CTM2-13-e1328-s001.docx]

**Supplementary files**

**
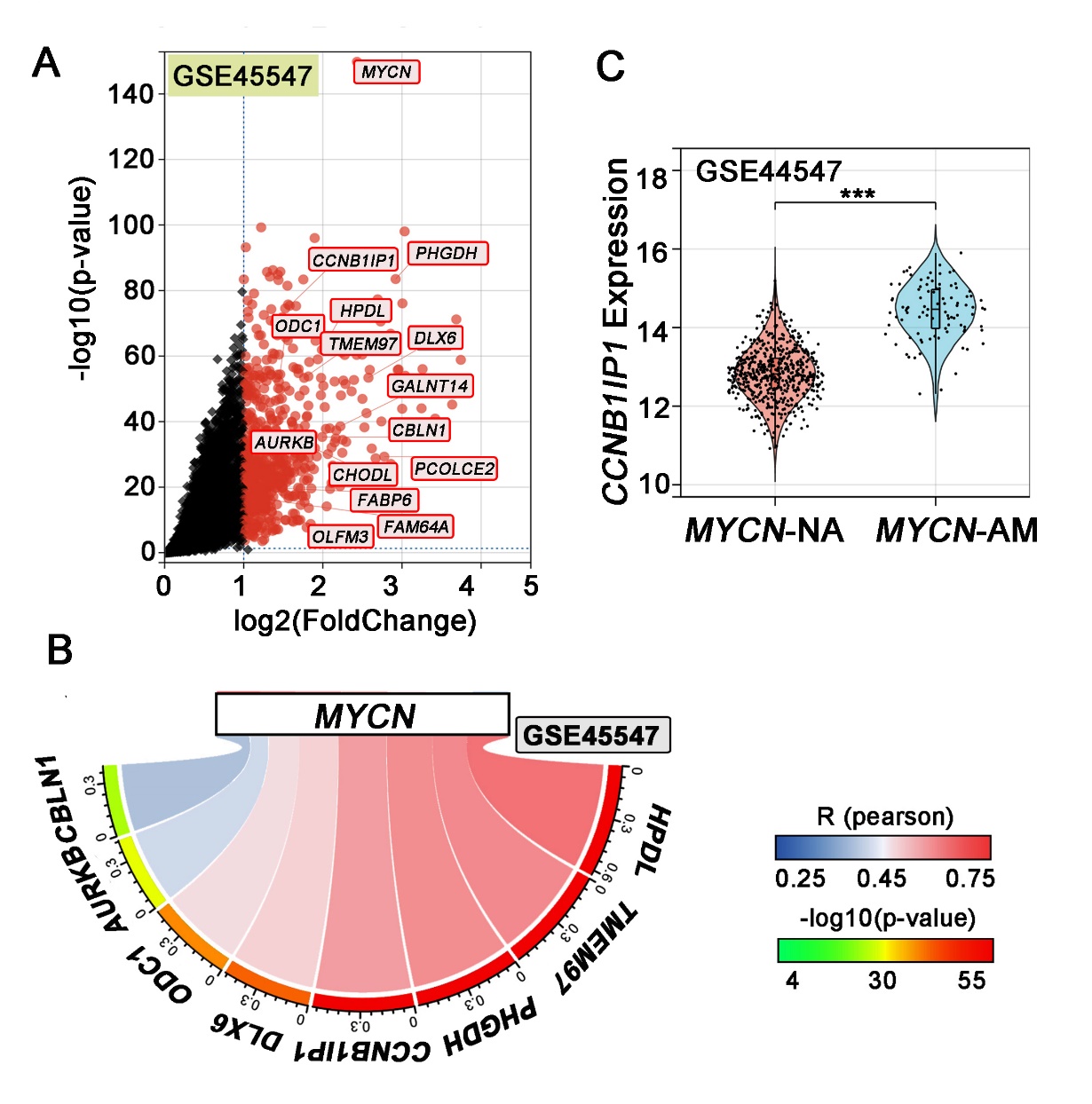
**

**Figure S1. *CCNB1IP1* overexpressed in *MYCN*-AM NB and correlated with MYCN expression.** (A) Differential genes between *MYCN*-AM and NA NB samples from GSE45547 dataset (log2FC > 1, FDR < 0.05). (B) Correlation between *CCNB1IP1* and *MYCN* transcription levels in NB cells from GSE45547 dataset. (C) *CCNB1IP1* expression in *MYCN*-NA and *MYCN*-AM NB patients in the GSE45547 dataset.

**
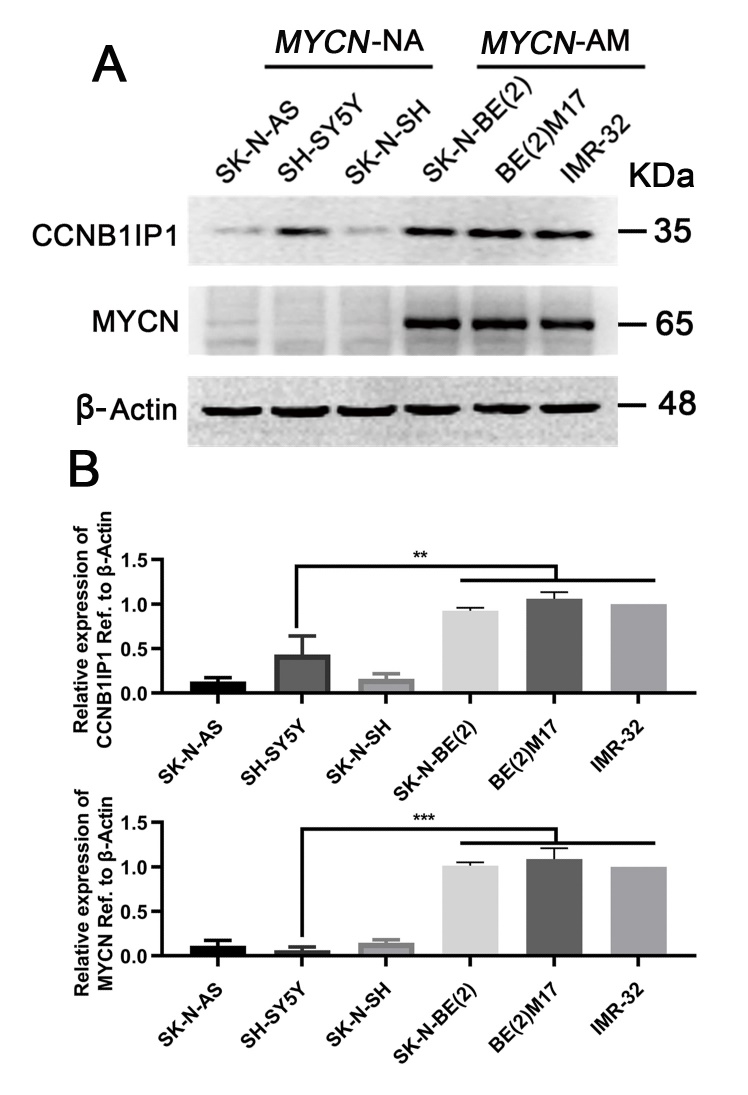
**

**Figure S2. The relative expression level of MYCN and CCNB1IP1 in MYCN-AM and NA NB cells.** (A) IB assay was performed. (B) Quantification of **A**. Data represent the mean ± SD of at least three independent experiments (^**^*P*＜0.01, ^***^*P*＜0.001).

**
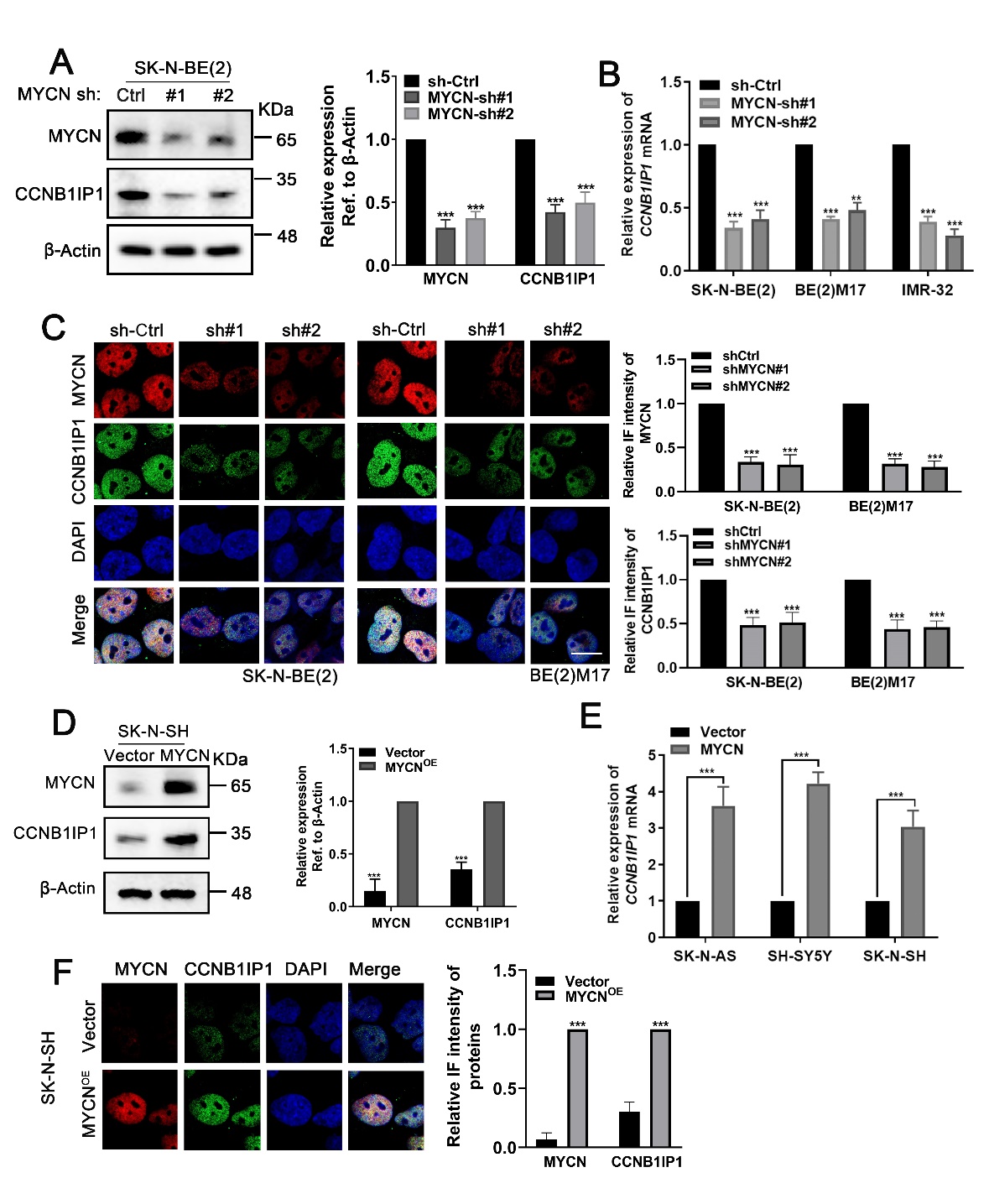
**

**Figure S3. MYCN regulates the expression and transcription of CCNB1IP1 in NB cells.** (A) IB assay of MYCN and CCNB1IP1 expression in SK-N-BE(2) cells was performed upon MYCN-shRNA knockdown for 48h. (B) qRT-PCR was performed to detect the mRNA expression of *CCNB1IP1* in SK-N-BE(2) cells treated as **A**. (C) Representative IF images of SK-N-BE(2) and BE(2)M17 cells. MYCN, red; CCNB1IP1, green; DAPI, blue. Scale bar = 10μm. (D) IB assay of MYCN and CCNB1IP1 expression in SK-N-SH-SY5Y cells upon MYCN overexpression. (E) qRT-PCR quantification of *CCNB1IP1* mRNA expression in SK-N-SH cells treated as in **D**. (F) Representative IF images. MYCN, red; CCNB1IP1, green; DAPI, blue. Scale bar = 10μm. Data represent the mean ± SD of at least three independent experiments (^***^*P*＜0.001).

**
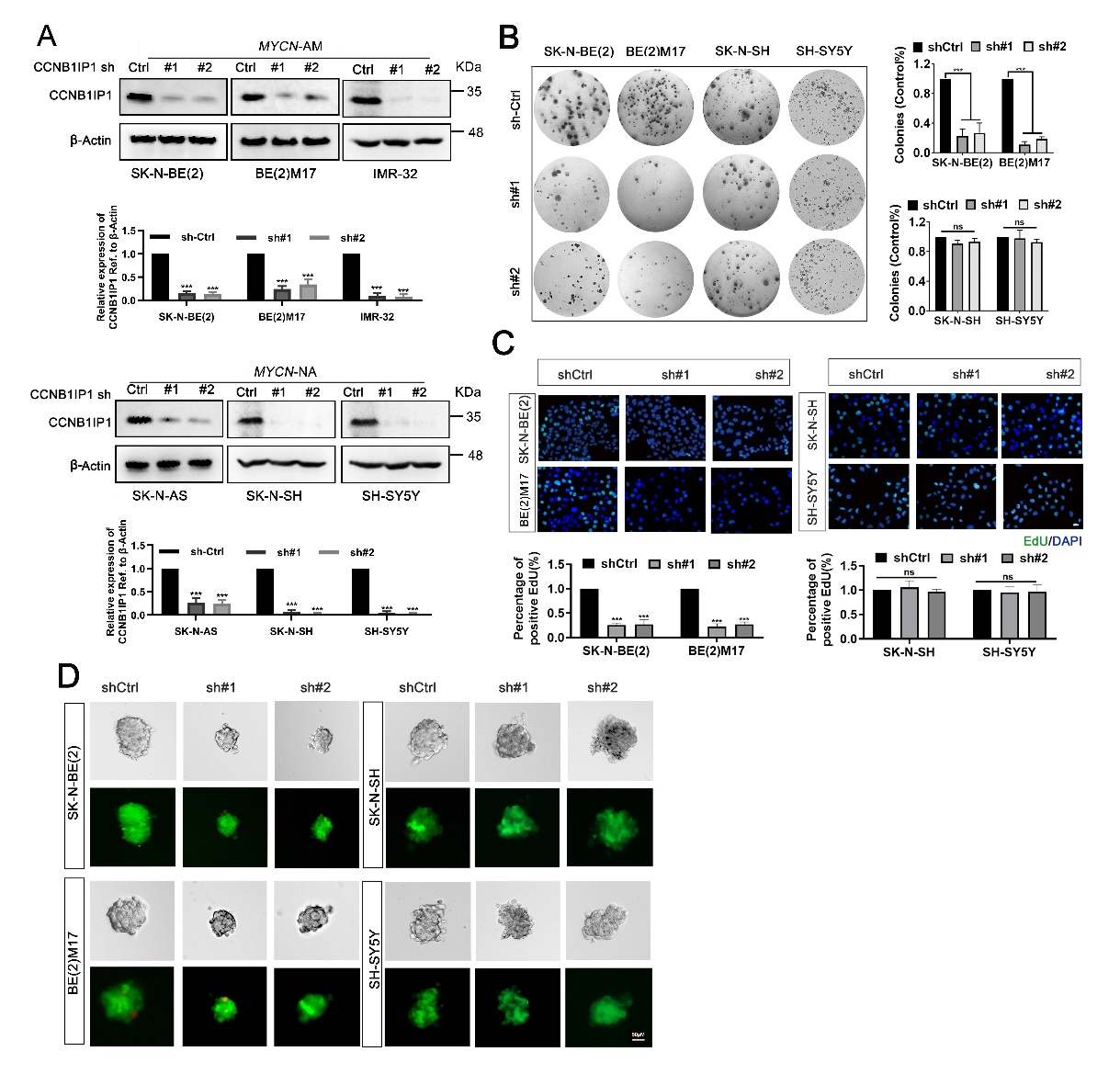
**

**Figure S4. Knockdown of CCNB1IP1 selectively inhibited the proliferation and growth of *MYCN*-AM NB cells.** Two shRNAs targeting CCNB1IP1 with different sequences were transfected into NB cells (SK-N-BE(2), BE(2)M17, IMR-32, SK-N-AS, SK-N-SH and SH-SY5Y cells) for 48h. (A) IB analysis of CCNB1IP1 protein levels was performed to detect the efficiency of shRNA. (B) Colony-formation assay. (C) EdU incorporation assay. Scale bar-10μM. (D) Tumor sphere formation experiment. Data represent at least three independent experiments (ns, no significant differences; ^**^*P*＜0.01 and ^***^*P*＜0.001).


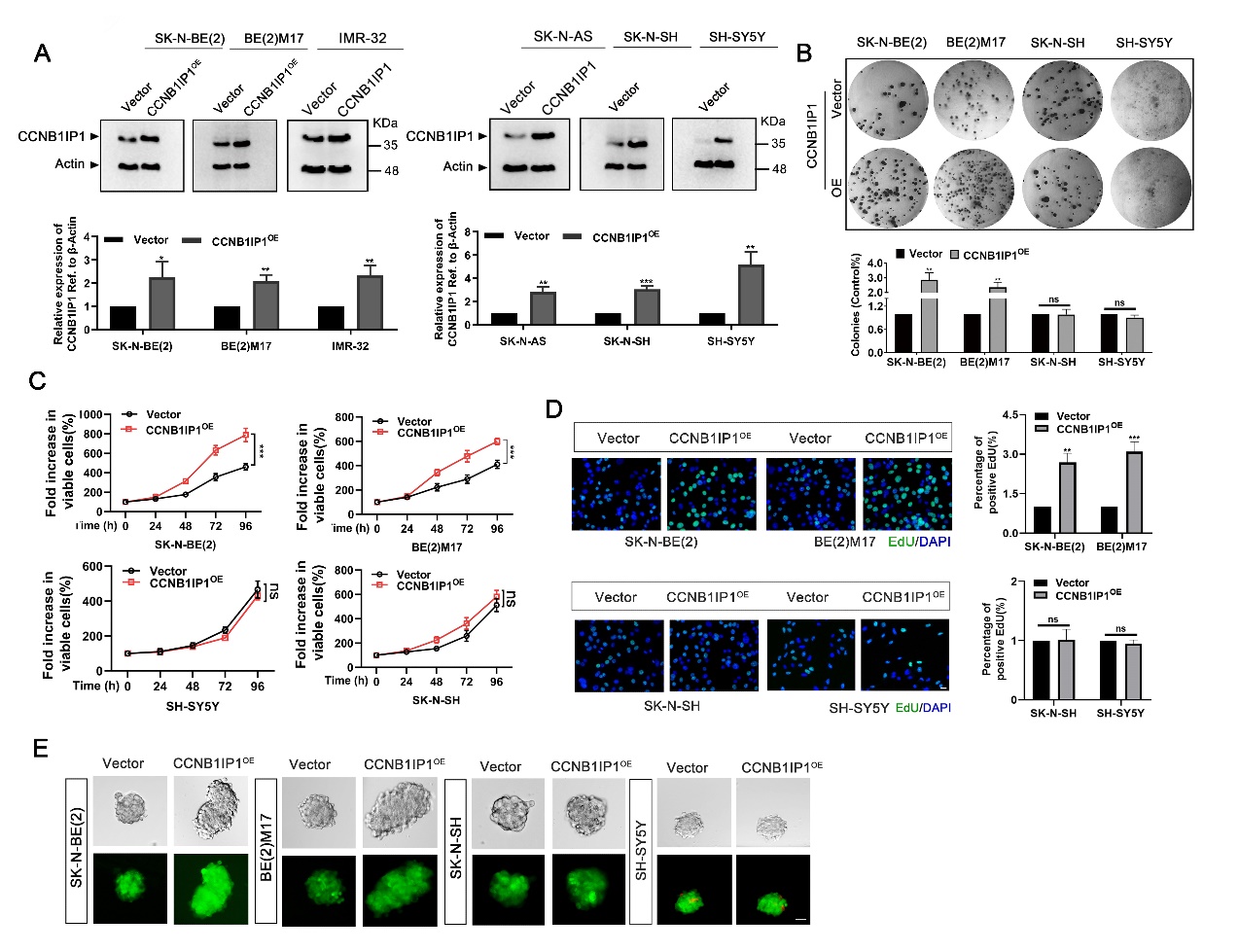


**Figure S5. Overexpression of CCNB1IP1 selectively promoted the proliferation and growth of MYCN-AM NB cells.** Empty vector or vectors encoding CCNB1IP1 were transfected into indicated NB cells for 48h. (A) IB analysis was performed to detect the CCNB1IP1 protein expression level. (B) Colony-formation assay. (C) MTT assay. (D) EdU incorporation assay. Scale bar-10μM. (E) Tumor sphere formation experiment. Data represent at least three independent experiments (ns, no significant differences; ^*^*P*＜0.05; ^**^*P*＜0.01 and ^***^*P*＜0.001).


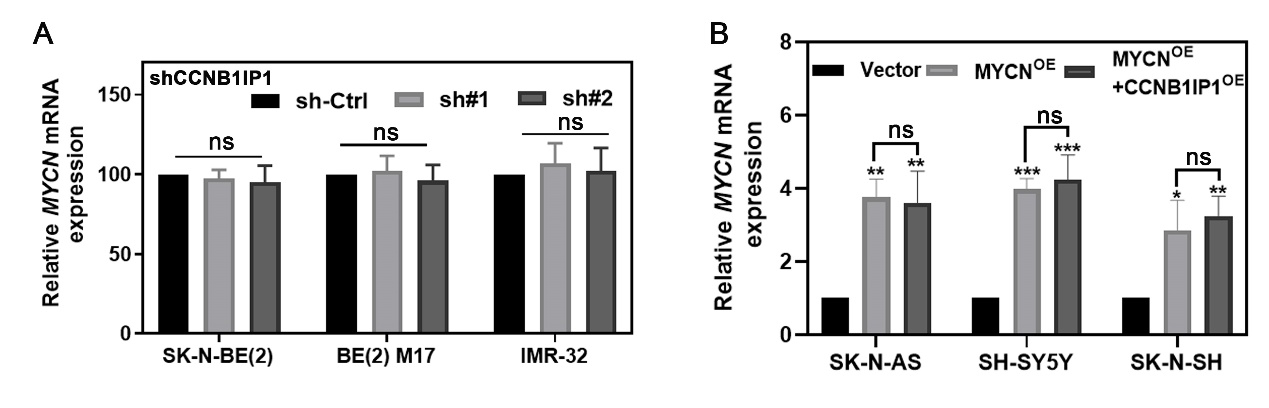


**Figure S6.** **Effect of CCNB1IP1 expression on *MYCN* mRNA levels.** Relative MYCN mRNA levels were detected by qRT-PCR assay. (A) *MYCN*-AM NB cells were transfected with CCNB1IP1 shRNAs. (B) MYCN-overexpressing *MYCN*-NA NB cells were transfected with CCNB1IP1 plasmid or empty vector. Data represent at least three independent experiments (ns, no significant differences; ^*^*P*＜0.05; ^**^*P*＜0.01 and ^***^*P*＜0.001).


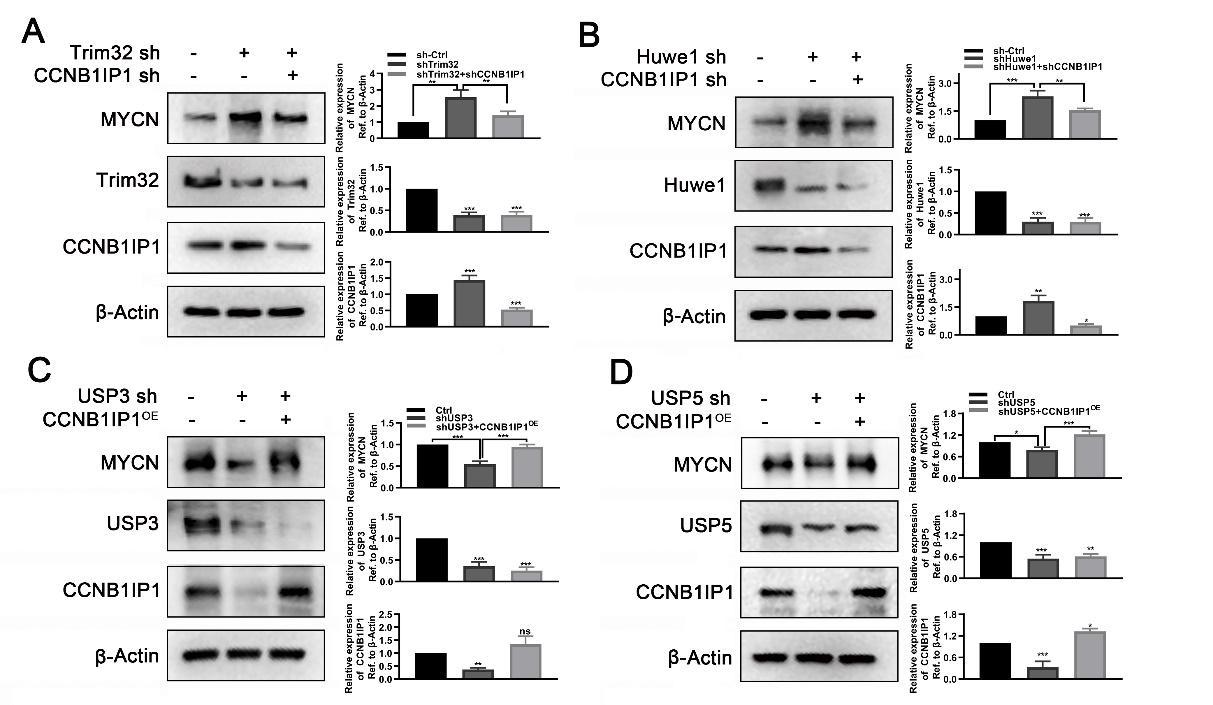


**Figure S7.** **CCNB1IP1 affects MYCN protein expression in a non-dependent manner on Trim32, Huwe1, USP3 and USP5.** IB analysis of Trim32, Huwe1, USP3, USP5, MYCN and CCNB1IP1 protein expression. BE(2)M17 cell infected with shTrim32 (A) shHuwe1 (B) alone or together with shCCNB1IP1 or infected with shUSP3 (C) shUSP5 (D) alone or together with CCNB1IP1 overexpression plasmid. All experiments were repeated at least three times (ns, no significant differences; ^*^*P*＜0.05; ^**^*P*＜0.01 and ^***^*P*＜0.001).

**
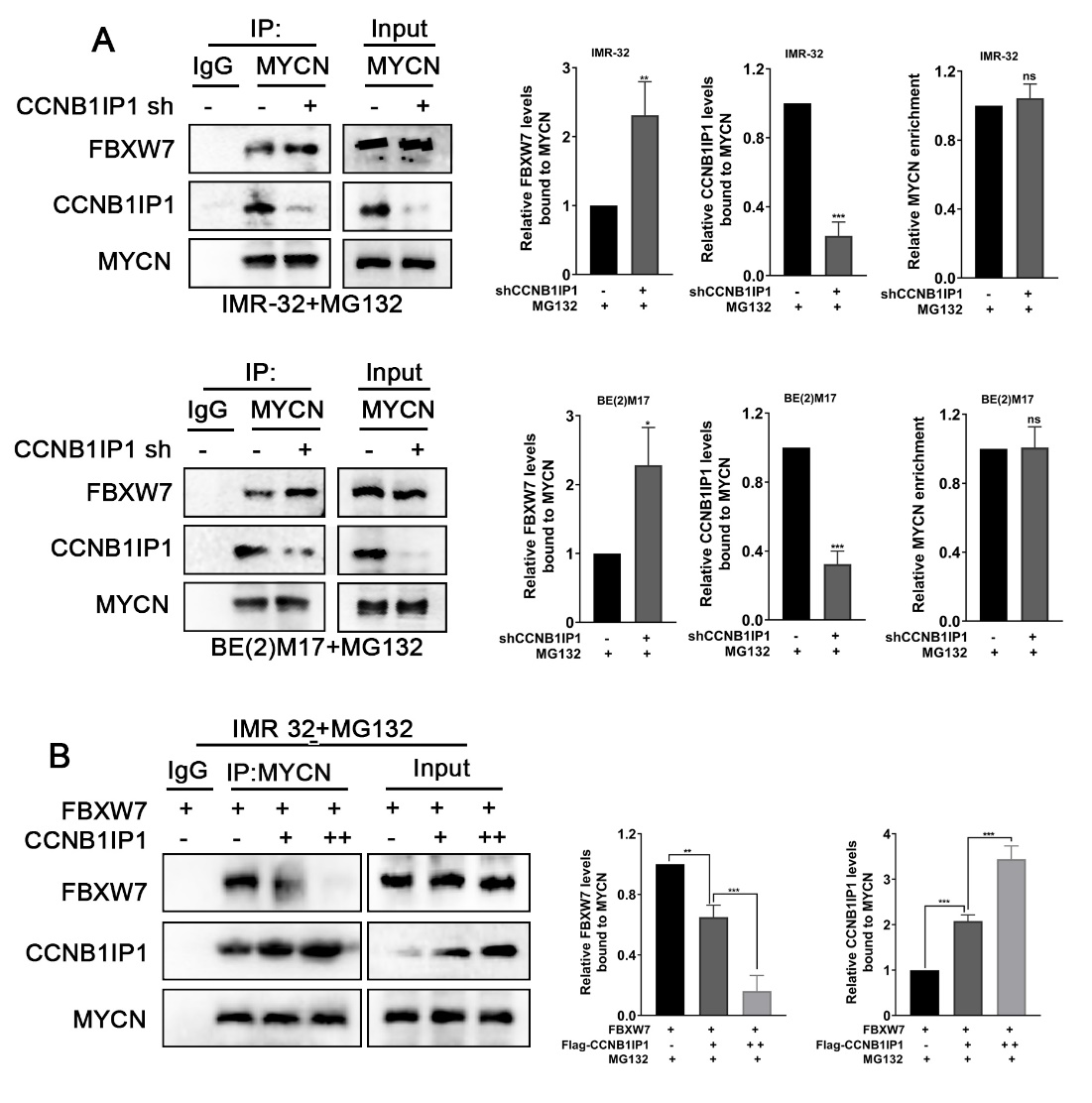
**

**Figure S8.** **CCNB1IP1 competing with FBXW7 for MYCN binding.** IP assay was performed to detect the interference of knockdown of (A) or exogenously expressed (B) CCNB1IP1 with the interaction between Fbxw7 and MYCN. IP assay was performed using anti-MYCN antibody and IgG was used as a negative control. All experiments were repeated at least three times (ns, no significant differences; ^*^*P*＜0.05; ^**^*P*＜0.01 and ^***^*P*＜0.001).
