# Supplementary material for: CCNB1IP1 prevents ubiquitination‐mediated destabilization of MYCN and potentiates tumourigenesis of MYCN‐amplificated neuroblastoma
Source: Clin Transl Med. 2023 Jul 17;13(7):e1328. doi: 10.1002/ctm2.1328 (PMC10352605; doi:10.1002/ctm2.1328)
Supplement: Supplementary file 2 — Tables S1–S5 [file CTM2-13-e1328-s002.docx]

**Table S1. The primer sequences for qRT-PCR.**

| Antibody | Source | Application and dilution | | | | |
| --- | --- | --- | --- | --- | --- | --- |
|  |  | WB | IF | IHC | IP | CHIP |
| CCNB1IP1 | Huabio | 1:1000 | 1:200 | 1:200 | - | - |
| MYCN | CST | 1:1000 | - | 1:200 | 1:200 | 1:50 |
|  | Invitrogen | - | 1:200 | - | - | - |
| HA | Abways | 1:1000 | - | - | 1:100 | - |
| His | Abways | 1:1000 | - | - | - | - |
| Flag | Abways | 1:1000 | - | - | 1:100 | - |
| Myc | Abways | 1:1000 | - | - | - | - |
| β-Actin | Abways | 1:3000 | - | - | - | - |
| FBXW7 | Proteintech | 1:1000 | - | - | - | - |
| USP3 | Proteintech | 1:1000 | - | - | - | - |
| USP5 | Proteintech | 1:1000 | - | - | - | - |
| Trim32 | Proteintech | 1:1000 | - | - | - | - |
| Ubiqiutin | Proteintech | 1:1000 | - | - | - | - |

**Table S2. The primer sequences for qRT-PCR.**

| Gene | Forward primer (**5**′ - 3′) | Reverse primer (**5**′ - 3′) |
| --- | --- | --- |
| β-Actin | CCTGGCACCCAGCACAAT | GGGCCGGACTCGTCATAC |
| CCNB1IP1 | ACTCAGCAAATACAAAGCAAGG | GCCTTCATGGTTAGCAATAGTG |
| MYCN | ATGAAGAGGAAGAAATCGACGT | CTTTATCTTCTTCTGTGGGGGT |

**Table S3. The sequences used in the dual luciferase reporter experiment**

| >NC_000014.9:c20335279-20333280 Homo sapiens chromosome 14, GRCh38.p13 Primary Assembly  GCTGTGATGTTCCAAGGCGTGGCAGGAAATCACATGGCCAGGATGCTGAGACTGCTAACATGCTATCCTTAAGTCTCTTTGCCTTTTCTTTAAAGCCACTAGTTCCCCTCCCATGATAACCTATTAATCCATGAATAGATTAATTTATTTATGAGGGCAGAACCCTCATGATCCAGTCACCTCTTGAAGGCCCCACTTCTAATGAGGATTAAGTTTCAGCCACTTTAGGGATTAAGTTTCAACATGAATTTTGTAAAAGGTGAAAGATTTATGCGATATGAAGAGAAACTAGAGTGACAACATGAATTTTGAAGGGGACAGTCAAACCATAGCAACTGGGATCTGAATCCTGACTTTACCACTTGCTAAATAGACAAATTCCTTAATCTCTATAACTCTGTTTCCTTTTTTTTTTTTTTTTTTTTGGTGAAATAGTTATAATGAAAGTGCTTACATTCTTGGGTTAATGTGAAGATTAAACAAGGTAATGAATATTACATTTCTTGAATATTTATAGTAAACATTCAAGAAATGTTTTGACTCTAGAAGTTACAATAAATCAATTATGAAATAACAAATTAGCATTTGATGCTAAATGTTGGGAAATTTGGGGCAGGTAAACTCTAAAGTGGATTGGAATAGGTAATTTCTAGTAAGGTGGAAGCAGGGTGGATAGGATGAAATGATTAAAAAATGAGAACTACTATGAGTTAGAGTTGTATCATTTCTCCATATAACTTGAATCTTTATACTTCAAACATTTGCACATTACATGTATATTTGTACATATAACACATACTTGTATATATATTCCCTATTACTATAATAGTATTTATTAAGAATAATGATGAATATTGTGCCGGGGAGTCCTCTAATTATGGCTTAAAAAGAAATCTTTCAGCTTGGGCAACATGGCGAAACCCCATCTCTACAAAAAAATACAAAAATTAGCTGGGTGTGATGGTGCACGCCTGTAGTCCCAGCTACTTACTGAGGCAGGAGATCGCTTGAACCTGGGAGGTCAAGGCTACAGTGAGTCGAGATTTCACCACTGCACTCCAGCCTGGGACACGAAGTGAGACCCTGTTTCAAAAAAAAAAAAAATTTAACAGAGTAAGAACCTATACTTTAACTGGAGGGCGATGTGAGGAGTCAAAATAATTGATTAATCAATTATGTCCTCAGCATTGAAAGTCATTGTTTTTGTCTGGAGATGTGTAAAACGTACACTTTGATCTCCAGTTGTTTATAGTCTACTTGGGAATATAAATCTAACAAGAAATATGTAATAACAATAATAACAGCTAACATTGTTGAGTACTGTGTACCGGGCATTGTACTGAGTACATTATATAAATTATTTTACTTAATCCTCATAACCATCAGAGTAGGAAGATTTCATCTTTTTTCCCTCTTTTTATAGTTGGAACTGAGGCTTAGATGTGTGCAAATAAGTGGCAAGGTTCAGATTCAAAATCAGCTAAATTTTATTTGAAGCCTAAAACACTTAACCCACCTACCTTTTATCTACTAAGCCTGTTAGTTCAAAGATTACCTGAAGGTAAATTTTATCAGGCCAGCTTTGGATCTGGGGGGAAAAAATGAAGGTAAATTTTAACTTGAATCGATCTAGACTGTTTATAATGGAATCTGTTGCCTAGCATAAGTTAAGCATGTTGAGGTCACAAAGGATAAGAAATTACCCTTAAGGGATGAAAATTCTTTAGAATAGAATGCAGTAGGCCCACGCAAACAACAACGTGTATAAATAGCTTAAATTAGCCTGTTTTTGCCTAAGTACATTAACTCAAAAGGAAACTCGGTTTAAGAAGTTCACTCCAAAAATAGTGCAAGAAGCATCACTGGTCCATAAAGAGGGGTGGAGTGGGTTACGTCATTTCAGCACCTTTTAAGGTAAAGGAGGAGGGAATCCTCTTGAACTCATTAGCCAATCAGGTTCTGAAGACACTTCCCAAGGCGACTTCCTGTCTCTCCACTTT |
| --- |

**Table S4. The primer sequences for ChIP-qPCR.**

| Fragments | Forward primer (**5**′ - 3′) | Reverse primer (**5**′ - 3′) |
| --- | --- | --- |
| NR | GGACACGAAGTGAGACCCTGTTTC | CCTCACATCGCCCTCCAGTTAAAG |
| PR | CAAGGCGTGGCAGGAAATCA | TTAATCCTCATTAGAAGTGGGGCCT |

**Table S5. The shRNA sequences.**

| ShFBXW7 | CCGGTTCAACAAGAACTTCGTAATTCTCGAGAATTACGAAGTTCTT |
| --- | --- |
| ShTrim32 | GTCCAATAGTCAAGTGGTAGAGGAGCAGA |
| ShHuwe1 | CGACGAGAACTAGCACAGAAT |
| ShUSP3 | AGTTTATCCGATCCAGCTT |
| ShUSP5 | CGA​GGA​GAA​GTT​TGA​ATT​A |
